# Supplementary material for: Active-site loop variations adjust activity and selectivity of the cumene dioxygenase
Source: Nat Commun. 2021 Feb 17;12:1095. doi: 10.1038/s41467-021-21328-8 (PMC7889853; doi:10.1038/s41467-021-21328-8)
Supplement: Supplementary file 1 — Supplementary Information [file 41467_2021_21328_MOESM1_ESM.pdf]

Active-site loop variations adjust activity and selectivity of the cumene dioxygenase

Supplementary Information

Peter M. Heinemann[a], Daniel Armbruster[a] and Bernhard Hauer [a]*

[a] Institute of Biochemistry and Technical Biochemistry, Department of Technical Biochemistry, University of Stuttgart, Allmandring 31, 70569 Stuttgart (Germany)

* Corresponding Author

E-mail address: bernhard.hauer@itb.uni-stuttgart.de

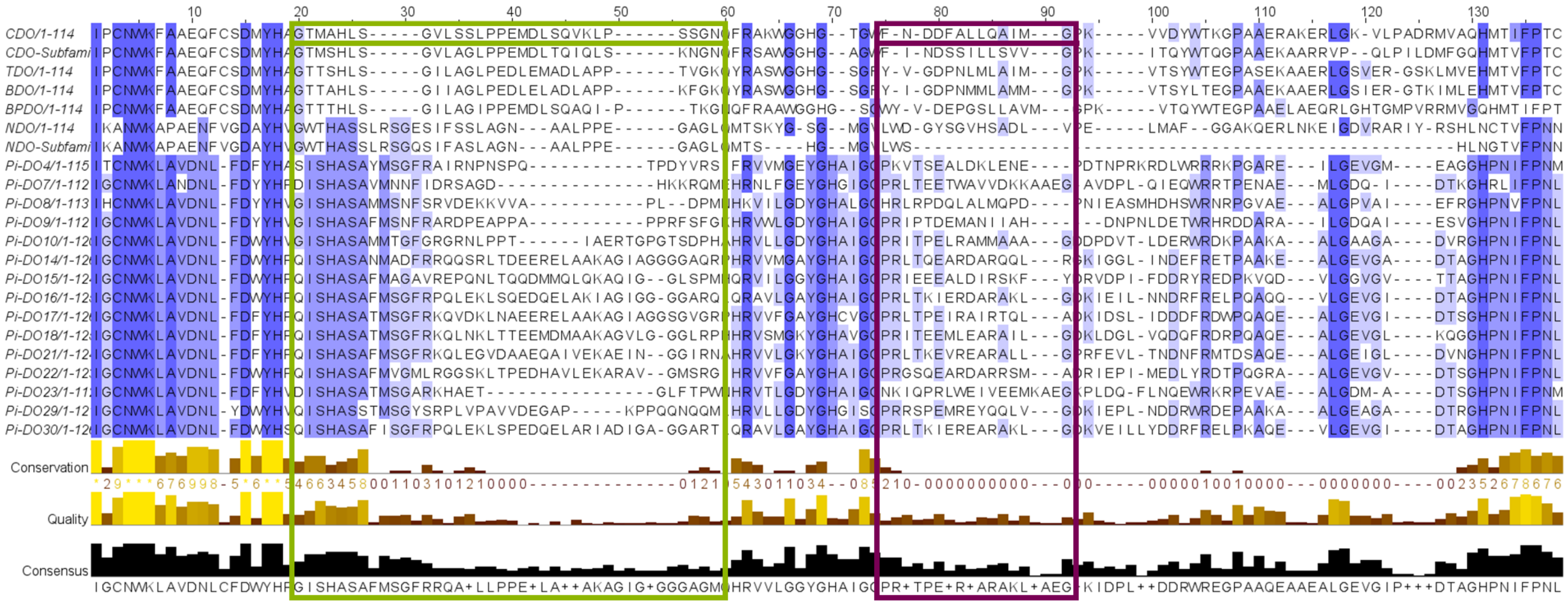

Supplementary Fig. 1: Excerpt of the sequence alignment of α-subunits of the CDO from *Pseudomonas fluorescens* IP01 with the CDO subfamily, biphenyl dioxygenase (BPDO) from *Paraburkholderia xenovorans* (LB400), toluene dioxygenase (TDO) from *Pseudomonas putida* F1, benzene dioxygenase (BDO) from *Pseudomonas putida* ML2, naphthalene dioxygenase (NDO) from *Pseudomonas* sp. NCIB 9816-4, the NDO subfamily and 15 α-subunits from the genome of *Phenylobacterium immobile* E (Pi-DO). The degree of conservation is shown from low (white) to high (blue) as well as in the conservation line. The alignment quality, consensus sequence and occupancy are shown in the bottom lines. Loop 1 (green), loop 2 (purple) and the corresponding residues of the other ROs are highlighted by frames in the corresponding colors.

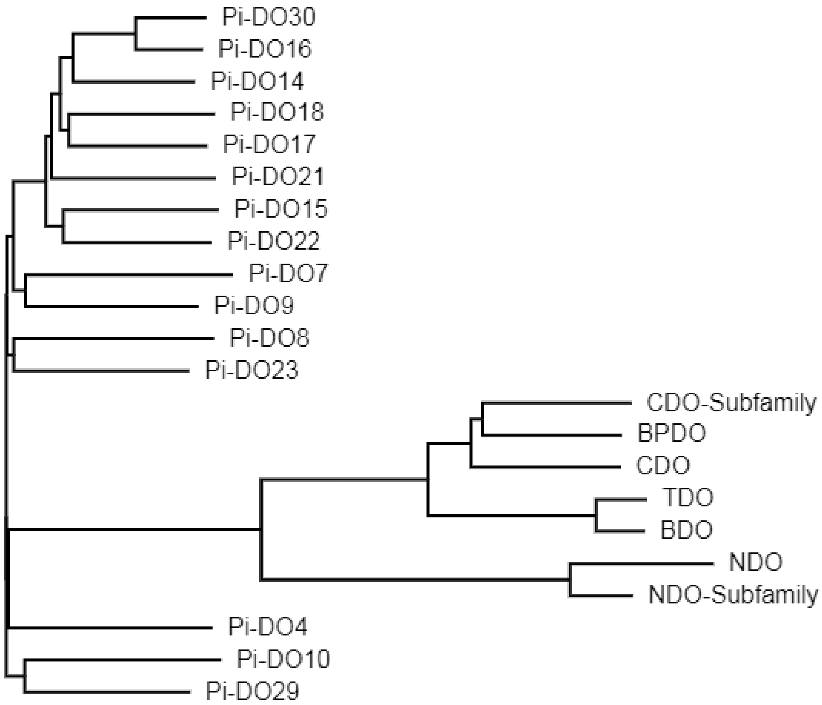

Supplementary Fig. 2: Phylogenetic tree of the aligned α-subunits of the oxygenases of CDO, CDO subfamily, BPDO, TDO, BDO, NDO, NDO-subfamily and from *Phenylobacterium immobile* E (Pi-DO).

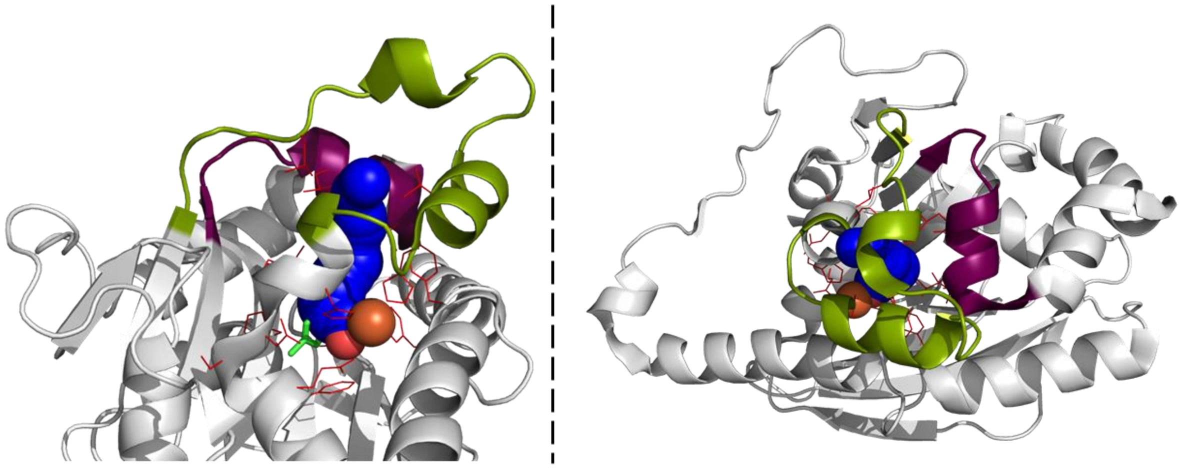

Supplementary Fig. 3: CAVER analysis of the crystal structure of the α-subunit of the CDO oxygenase from *Pseudomonas fluorescens* IP01 (PDB entry 1wql). Side view (left structure) and top view (right structure) of loop 1 (green) and loop 2 (purple) in the crystal structure of one α-subunit (grey) of the CDO oxygenase (PDB entry 1wql). The simulated tunnel (blue), the catalytically active iron (orange sphere), molecular oxygen (red spheres) and active site residues (red sticks) are shown as well as the docked substrate *R*-limonene (green sticks). Iron was selected as starting point with a tunnel radius of 1.2 Å.

Supplementary Table 1: Conversions of styrene 1, (*R*)-limonene 2 and 2-phenylpydridine 3 with the Wild-type and the variants obtained from the alanine scan of the CDO. Biotransformations were performed in technical triplicates and standard deviations (calculated using Excel version 2016) are indicated. Pf: combined product formation in mM. Regioselectivity in %. Source data are provided as a Source Data file.

	Pf (mM)	Selectivity (%)		Pf (mM)	Selectivity (%)			Pf (mM)	Selectivity (%)	
		1a	1b		2a	2b	2c		3a	3b
Wild-type	6.68 ± 0.77	86.4	13.6	3.56 ± 0.44	98.8	0.7	0.5	0.64 ± 0.03	93.0	7.0
G236A	10.22 ± 1.07	28.4	71.6	3.77 ± 0.21	98.3	0.9	0.8	0.87 ± 0.03	91.7	8.3
T237A	8.63 ± 1.32	72.1	27.9	2.60 ± 0.14	97.1	1.4	1.5	0.90 ± 0.02	94.9	5.1
M238A	0 ± 0	0.0	0.0	0.29 ± 0.02	100.0	0.0	0.0	0 ± 0	0.0	0.0
A239G	10.07 ± 1.35	85.5	14.5	0.84 ± 0.03	98.8	1.2	0.0	0.13 ± 0.01	89.2	10.8
H240A	0 ± 0	0.0	0.0	0 ± 0	0.0	0.0	0.0	0 ± 0	0.0	0.0
L241A	10.26 ± 1.80	77.0	23.0	2.44 ± 0.26	99.3	0.4	0.4	1.41 ± 0.08	92.9	7.1
S242A	1.56 ± 0.26	84.4	15.6	0.37 ± 0.01	97.0	0.8	2.2	0.15 ± 0.01	92.3	7.7
G243A	0.33 ± 0.06	32.9	67.1	0.17 ± 0.00	97.0	0.0	3.0	0.07 ± 0.00	100.0	0.0
V244A	0.73 ± 0.11	45.9	54.1	0.22 ± 0.01	95.7	1.4	2.9	0.09 ± 0.01	89.9	10.1
L245A	4.94 ± 0.43	90.7	9.3	0.60 ± 0.06	97.3	0.9	1.9	0.16 ± 0.01	93.5	6.5
S246A	5.17 ± 0.07	89.9	10.1	1.23 ± 0.02	97.6	1.5	0.9	0.17 ± 0.01	93.7	6.3
S247A	2.81 ± 0.17	80.7	19.3	0.56 ± 0.08	96.1	1.5	2.4	0.15 ± 0.00	93.6	6.4
L248A	4.42 ± 0.21	61.1	38.9	1.19 ± 0.04	97.9	0.6	1.5	0.69 ± 0.06	92.7	7.3
P249A	8.11 ± 1.36	79.4	20.6	3.42 ± 0.02	98.8	0.5	0.7	0.79 ± 0.00	93.4	6.6
P250A	6.66 ± 0.30	80.8	19.2	2.81 ± 0.34	99.0	0.4	0.5	0.89 ± 0.02	93.1	6.9
E251A	7.00 ± 0.29	72.7	27.3	2.92 ± 0.15	99.2	0.3	0.6	0.68 ± 0.02	93.1	6.9
M252A	4.22 ± 0.28	77.3	22.7	3.10 ± 0.53	98.7	0.7	0.7	0.66 ± 0.02	93.2	6.8
D253A	3.09 ± 0.35	69.5	30.5	2.44 ± 0.14	99.1	0.2	0.7	0.41 ± 0.03	95.4	4.6
L254A	2.32 ± 1.95	76.3	23.7	1.81 ± 0.06	99.2	0.0	0.8	0.57 ± 0.04	95.1	4.9

S255A	5.41 ± 0.29	77.9	22.1	2.56 ± 0.08	98.7	0.6	0.6	0.80 ± 0.03	93.5	6.5
Q256A	6.53 ± 0.60	79.1	20.9	2.81 ± 0.16	99.0	0.6	0.4	0.71 ± 0.02	93.2	6.8
V257A	6.14 ± 0.54	69.6	30.4	2.77 ± 0.07	98.8	0.4	0.8	0.74 ± 0.05	95.3	4.7
K258A	1.32 ± 0.40	77.3	22.7	2.85 ± 0.07	98.7	0.6	0.7	0.46 ± 0.10	92.7	7.3
L259A	2.20 ± 0.31	93.8	6.2	2.72 ± 0.17	97.9	0.8	1.2	0.53 ± 0.11	92.8	7.2
P260A	0 ± 0	0.0	0.0	1.78 ± 0.15	97.9	1.0	1.1	0.11 ± 0.01	84.0	16.0
S261A	0.94 ± 0.25	74.9	25.1	2.50 ± 0.13	98.3	0.8	0.9	0.33 ± 0.02	89.7	10.3
S262A	1.73 ± 0.04	87.2	12.8	0.29 ± 0.01	100.0	0.0	0.0	0.53 ± 0.01	92.9	7.1
G263A	0.56 ± 0.11	0.0	100.0	3.25 ± 0.64	99.3	0.7	0.0	0.25 ± 0.00	87.5	12.5
N264A	3.04 ± 0.31	87.4	12.6	2.66 ± 0.28	100.0	0.0	0.0	0.25 ± 0.01	92.5	7.5
F278A	14.24 ± 0.49	75.8	24.2	3.36 ± 0.15	99.3	0.5	0.1	2.67 ± 0.11	85.8	14.2
N279A	14.71 ± 1.38	73.7	26.3	2.88 ± 0.12	97.8	1.0	1.2	1.26 ± 0.11	92.2	7.8
D280A	16.21 ± 0.18	76.3	23.7	3.06 ± 0.10	99.2	0.7	0.1	1.28 ± 0.09	93.1	6.9
D281A	14.56 ± 0.94	12.6	87.4	1.29 ± 0.02	93.0	2.6	4.4	1.56 ± 0.03	89.2	10.8
F282A	12.31 ± 2.86	95.0	5.0	4.24 ± 0.17	100.0	0.0	0.0	9.99 ± 0.40	98.6	1.4
A283G	5.30 ± 0.70	70.5	29.5	2.89 ± 0.13	98.2	0.9	0.8	1.02 ± 0.06	93.1	6.9
L284A	5.04 ± 0.51	27.9	72.1	2.01 ± 0.24	61.6	20.1	18.3	1.32 ± 0.04	90.5	9.5
L285A	4.98 ± 0.85	15.2	84.8	0.16 ± 0.00	100.0	0.0	0.0	0.28 ± 0.02	83.3	16.7
Q286A	4.87 ± 0.84	51.1	48.9	1.32 ± 0.01	98.7	1.3	0.0	0.36 ± 0.02	90.7	9.3
A287G	6.42 ± 0.11	29.5	70.5	1.29 ± 0.10	97.8	2.2	0.0	0.38 ± 0.02	88.2	11.8
I288A	5.06 ± 1.12	82.2	17.8	0.45 ± 0.03	100.0	0.0	0.0	0.48 ± 0.01	93.5	6.5
M289A	5.63 ± 0.56	53.3	46.7	1.94 ± 0.09	54.1	6.2	39.8	10.05 ± 0.13	93.3	6.7
G290A	6.57 ± 0.37	68.8	31.2	2.75 ± 0.26	98.8	0.6	0.6	0.35 ± 0.02	90.1	9.9

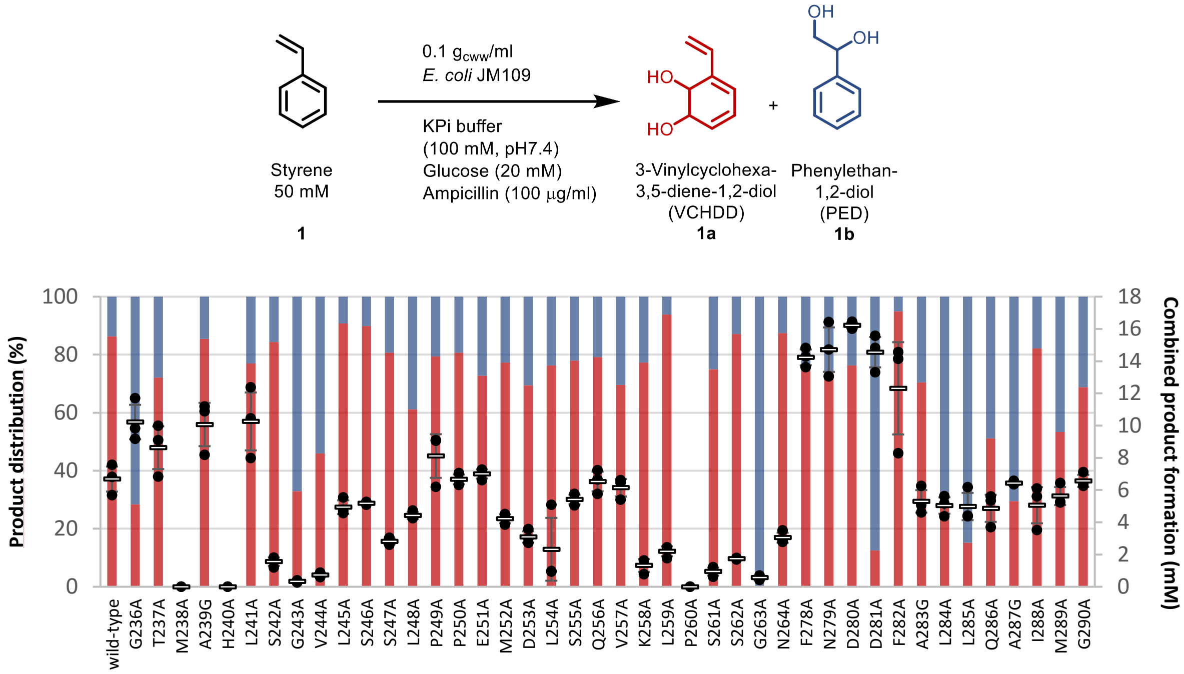

Supplementary Fig. 4: Product distribution and formation of the two products 3-vinylcyclohexa-3,5-diene-1,2-diol 1a (VCHDD, red, bottom bars) and phenylethan-1,2-diol 1b (PED, blue, top bars) for the biotransformation of styrene with the wild-type and variants obtained from the alanine scan of loop 1 and 2 of the CDO. The reactions were performed in technical triplicates (black dots) with average values (horizontal bar) and standard deviations (calculated using Excel version 2016) indicated. Source data are provided as a Source Data file.

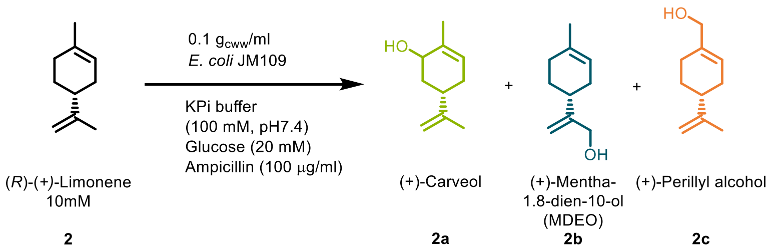

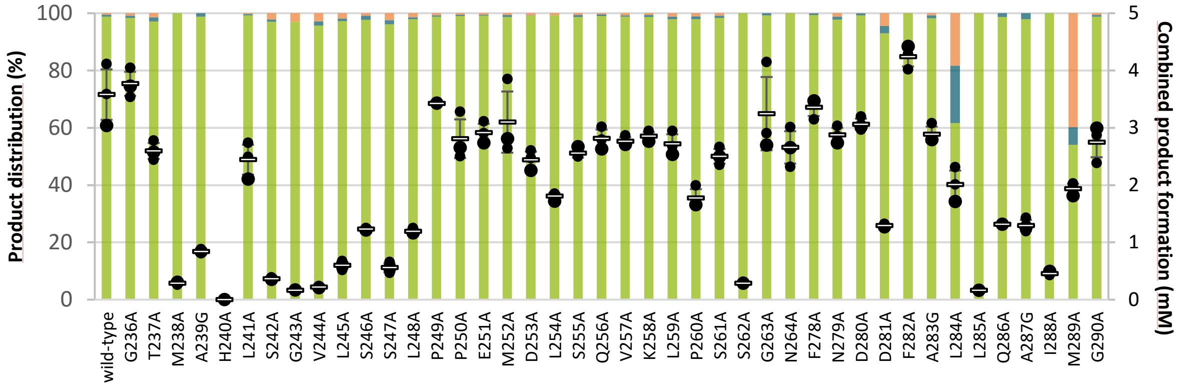

Supplementary Fig. 5: Product distribution and formation of the products (+)-carveol 2a (green, bottom bars), (+)-mentha-1.8-dien-10-ol 2b (MDEO, blue, middle bars) and (+)-perillyl alcohol 2c (orange, top bars) for the biotransformation of (*R*)-limonene 2 with the wild-type and variants obtained from the alanine scan of loop 1 and 2 of the CDO. The reactions were performed in technical triplicates (black dots) with average values (horizontal bar) and standard deviations (calculated using Excel version 2016) indicated. Source data are provided as a Source Data file.

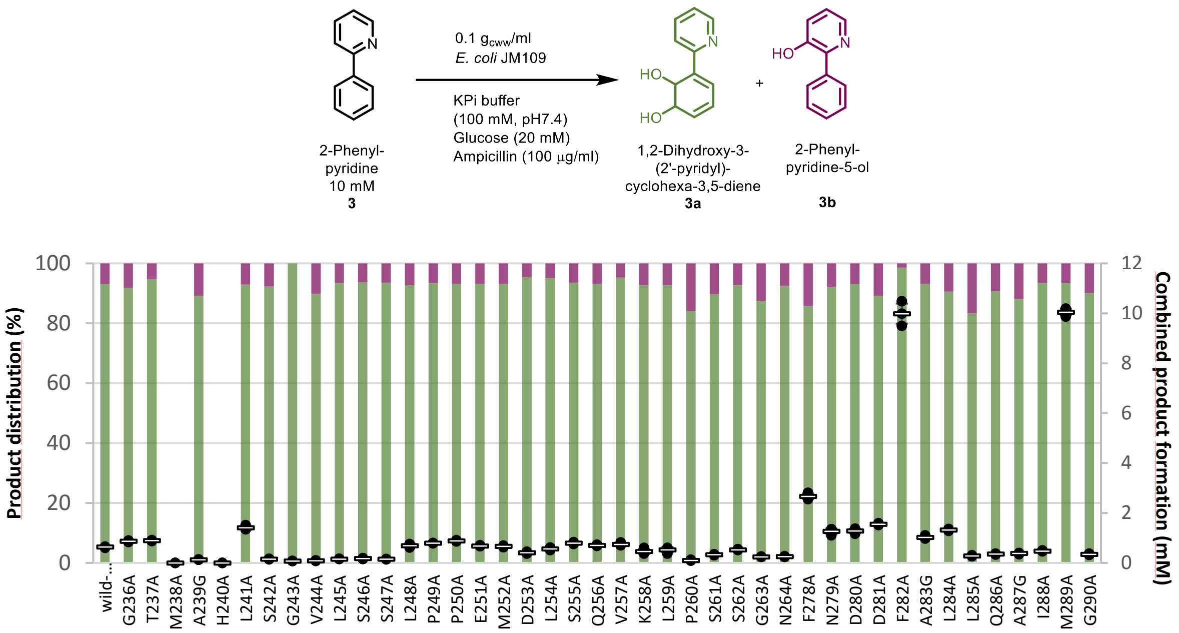

Supplementary Fig. 6: Product distribution of the products 1,2-dihydroxy-3-(2'-pyridyl)cyclohexa-3,5-diene 3a (green, bottom bars) and 2-phenylpyridine-5-ol 3b (purple, top bars) for the biotransformation of 2-phenylpyridine 3 with the wild-type and variants obtained from the alanine scan of loop 1 and 2 of the CDO. The reactions were performed in technical triplicates (black dots) with average values (horizontal bar) and standard deviations (calculated using Excel version 2016) indicated. Source data are provided as a Source Data file.

Supplementary Table 2: Conversion of styrene 1, (*R*)-limonene 2 and 2-phenylpydridine 3 with the Wild-type and selected saturation variants. Biotransformations were performed in technical triplicates and standard deviation (calculated using Excel version 2016) are indicated. Pf: combined product formation in mM. Regioselectivity in %. Source data are provided as a Source Data file.

	**Pf (mM)**	**Selectivity (%)**		**Pf (mM)**	**Selectivity (%)**			**Pf (mM)**	**Selectivity (%)**	
		**1a**	**1b**		**2a**	**2b**	**2c**		**3a**	**3b**
Wild-type	6.68 ± 0.77	86.4	13.6	3.58 ± 0.44	98.8	0.7	0.5	0.64 ± 0.03	93.0	7.0
F278V	0.24 ± 0.07	100.0	0.0	5.31 ± 0.79	100.0	0.0	0.0	1.57 ± 0.04	94.1	5.9
F282V	1.31 ± 0.06	69.4	30.6	5.15 ± 0.28	100.0	0.0	0.0	1.28 ± 0.02	97.0	3.0
F282T	0.05 ± 0.00	100.0	0.0	2.78 ± 0.24	100.0	0.0	0.0	9.82 ± 0.20	97.3	2.7
L284G	0.31 ± 0.08	84.7	15.3	2.08 ± 0.19	30.8	19.4	49.7	0.74 ± 0.04	91.4	8.6
Q286F	0.65 ± 0.05	67.5	32.5	1.86 ± 0.16	100.0	0.0	0.0	0.40 ± 0.05	93.8	6.2
I288S	0.02 ± 0.03	41.6	58.4	1.31 ± 0.41	69.9	3.5	26.5	1.11 ± 0.03	93.3	6.7
I288T	0.10 ± 0.01	73.9	26.1	2.14 ± 0.20	96.4	0.1	3.4	1.96 ± 0.02	93.6	6.4
M289K	0.09 ± 0.01	0.0	100.0	0.06 ± 0.00	100.0	0.0	0.0	0.15 ± 0.01	86.7	13.3

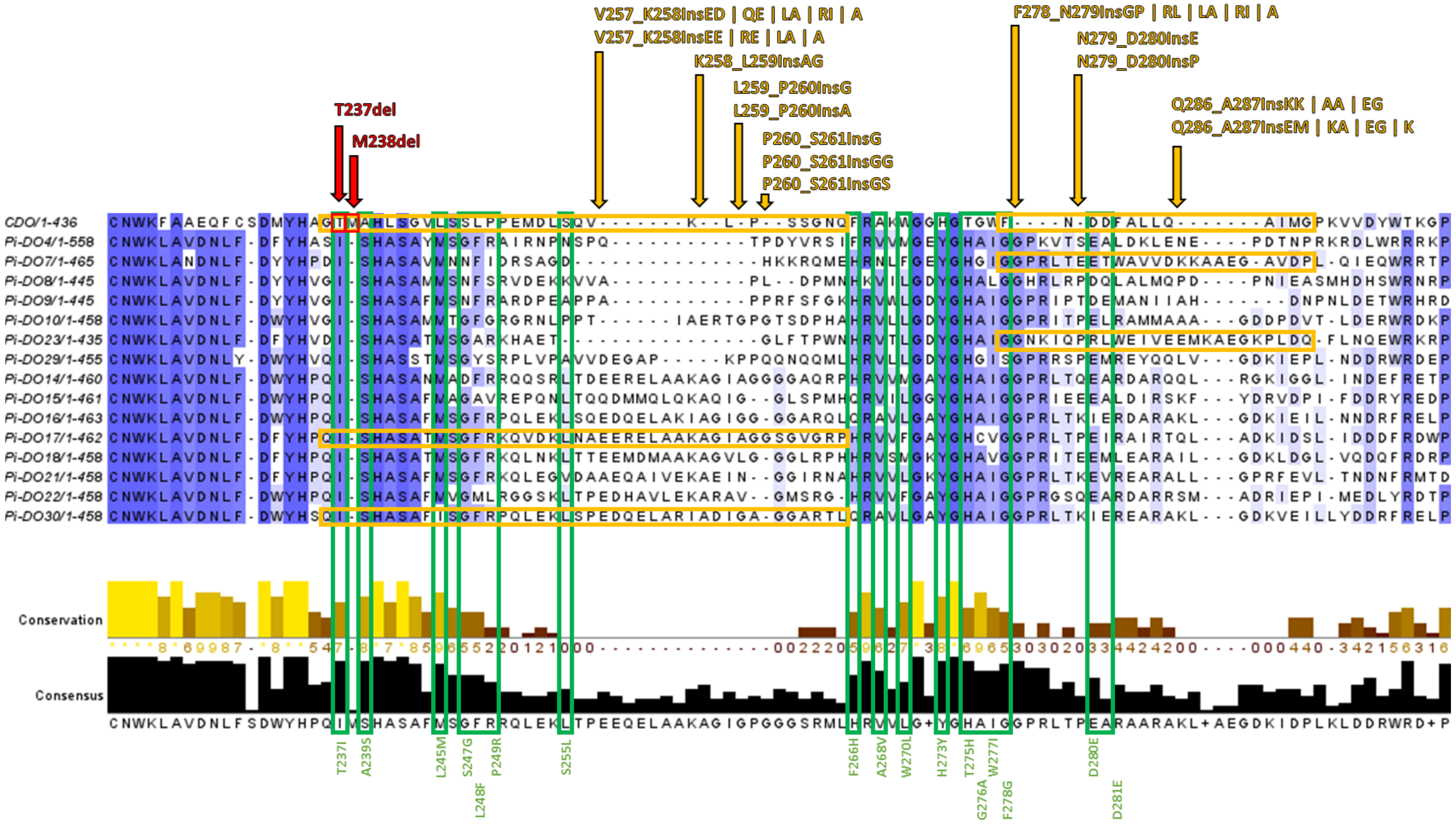

Supplementary Fig. 7: Excerpt of the sequence alignment of CDO with α-subunits of the oxygenases from *Phenylobacterium immobile* E and variant selection for the adaption library. Single point mutations are indicated in green, single point deletions in red. Introduced sequence-alignment based insertions are highlighted in yellow, the loops 1 and 2 of the CDO together with the longest corresponding loops from oxygenases originated from *Phenylobacterium immobile* E are framed in yellow. Slashes in the variant designation represent stepwise insertions

Supplementary Table 3: Conversions of styrene 1, (*R*)-limonene 2 and 2-phenylpydridine 3 with the wild-type, and deletion and single-point mutation variants based on loops in oxygenases from *Phenylobacterium immobile* E. Biotransformations were performed in technical triplicates and standard deviation (calculated using Excel version 2016) are indicated. Pf: combined product formation in mM. Regioselectivity in %. Source data are provided as a Source Data file.

	Pf (mM)	Selectivity (%)		Pf (mM)	Selectivity (%)			Pf (mM)	Selectivity (%)	
		1a	1b		2a	2b	2c		3a	3b
Wild-type	6.68 ± 0.77	86.4	13.6	3.58 ± 0.44	98.8	0.7	0.5	0.64 ± 0.03	93.0	7.0
T237del	0 ± 0	0.0	0.0	0 ± 0	0.0	0.0	0.0	0 ± 0	0.0	0.0
M238del	0 ± 0	0.0	0.0	0.12 ± 0.00	100.0	0.0	0.0	0 ± 0	0.0	0.0
T237I	7.99 ± 0.13	78.5	21.5	0.44 ± 0.03	100.0	0.0	0.0	0.06 ± 0.01	100.0	0.0
A239S	6.89 ± 0.21	69.8	30.2	0.86 ± 0.02	97.4	0.8	1.8	0.55 ± 0.02	91.0	9.0
L245M	0 ± 0	0.0	0.0	0.10 ± 0.00	100.0	0.0	0.0	0 ± 0	0.0	0.0
S247G	0 ± 0	0.0	0.0	0 ± 0	0.0	0.0	0.0	0 ± 0	0.0	0.0
L248F	0 ± 0	0.0	0.0	0 ± 0	0.0	0.0	0.0	0 ± 0	0.0	0.0
P249R	0 ± 0	0.0	0.0	0 ± 0	0.0	0.0	0.0	0 ± 0	0.0	0.0
S255L	5.35 ± 0.10	71.8	28.2	0.58 ± 0.02	96.7	2.3	1.0	0.05 ± 0.01	81.4	18.6
F266H	8.14 ± 0.42	75.8	24.2	1.38 ± 0.02	98.0	0.9	1.1	0.11 ± 0.01	86.6	13.4
A268V	4.18 ± 0.07	73.6	26.4	0.84 ± 0.06	97.4	1.9	0.7	0 ± 0	0.0	0.0
W270L	2.52 ± 0.12	84.4	15.6	2.74 ± 0.41	99.1	0.4	0.5	0.22 ± 0.02	92.4	7.6
H273Y	0 ± 0	0.0	0.0	0.14 ± 0.00	100.0	0.0	0.0	0 ± 0	0.0	0.0
T275H	1.46 ± 0.07	84.4	15.6	1.39 ± 0.02	99.2	0.4	0.5	0.51 ± 0.01	95.4	4.6
G276A	0 ± 0	0.0	0.0	1.22 ± 0.06	100.0	0.0	0.0	0.19 ± 0.01	92.1	7.9
W277I	0 ± 0	0.0	0.0	0 ± 0	0.0	0.0	0.0	0 ± 0	0.0	0.0
F278G	0.93 ± 0.13	75.5	24.5	2.12 ± 0.05	96.7	0.9	2.4	0.49 ± 0.02	86.2	13.8
D280E	2.24 ± 0.04	89.1	10.9	3.21 ± 0.18	99.5	0.5	0.0	0.37 ± 0.01	93.4	6.6
D281E	14.25 ± 0.49	55.9	44.1	0.64 ± 0.03	100.0	0.0	0.0	0.07 ± 0.01	90.9	9.1

Supplementary Table 4: Conversions of styrene 1, (*R*)-limonene 2 and 2-phenylpydridine 3 with the wild-type and insertion variants based on loops in oxygenases from *Phenylobacterium immobile* E. Biotransformations were performed in technical triplicates and standard deviations (calculated using Excel version 2016) are indicated. Pf: combined product formation in mM. Regioselectivity in %. Source data are provided as a Source Data file.

	Pf (mM)	Selectivity (%)		Pf (mM)	Selectivity (%)			Pf (mM)	Selectivity (%)	
		1a	1b		2a	2b	2c		3a	3b
Wild-type	6.68 ± 0.77	86.4	13.6	3.58 ± 0.44	98.8	0.7	0.5	0.64 ± 0.03	93.0	7.0
V257_K258 insED	12.81 ± 1.50	79.7	20.3	7.81 ± 0.31	98.4	0.7	0.9	0.64 ± 0.05	92.8	7.2
V257_K258 insEDQE	4.42 ± 0.50	79.9	20.1	0.44 ± 0.01	97.3	1.2	1.5	0.08 ± 0.01	89.8	10.2
V257_K258 insEDQELA	5.25 ± 0.74	82.3	17.7	0.314 ± 0.00	95.0	3.2	1.8	0.09 ± 0.01	90.1	9.9
V257_K258 insEDQELA RI	8.78293 ± 0.86	81.0	19.0	2.75 ± 0.19	98.6	0.8	0.6	0.42 ± 0.02	94.9	5.1
V257_K258 insEDQELA RIA	8.61 ± 0.68	81.3	18.7	0.77 ± 0.02	100.0	0.0	0.0	0.38 ± 0.12	94.7	5.3
L259_P260 insG	6.68 ± 0.15	72.6	27.4	0.62 ± 0.01	97.0	0.0	3.0	0.12 ± 0.00	91.2	8.8
P260_S261 insG	11.23 ± 0.29	78.6	21.4	1.22 ± 0.01	98.7	1.3	0.0	0.10 ± 0.01	92.5	7.5
V257_K258 insEE	13.81 ± 0.15	46.5	53.5	1.37 ± 0.04	97.2	0.9	2.0	0.10 ± 0.01	91.7	8.3
V257_K258 insEERE	1.86 ± 0.11	64.0	36.0	0.12 ± 0.01	100.0	0.0	0.0	0 ± 0	0.0	0.0
V257_K258 insEERELA	4.88 ± 0.07	77.2	22.8	0.43 ± 0.03	92.1	3.9	4.0	0.12 ± 0.01	90.2	9.8
V257_K258 insEERELA A	13.14 ± 0.82	84.9	15.1	6.39 ± 0.23	98.4	0.7	1.0	1.97 ± 0.13	96.2	3.8
K258_L259 insAG	17.75 ± 0.91	56.3	43.7	0.35 ± 0.01	100.0	0.0	0.0	0.26 ± 0.01	91.7	8.3
L259_P260 insA	22.06 ± 1.51	76.4	23.6	5.55 ± 0.46	96.3	1.7	1.9	1.05 ± 0.07	92.5	7.5
P260_S261 insGS	0 ± 0	0.0	0.0	0.092 ± 0.01	100.0	0.0	0.0	0 ± 0	0.0	0.0
P260_S261 insGG	10.55 ± 0.27	66.2	33.8	0.63 ± 0.27	100.0	0.0	0.0	0.07 ± 0.01	92.3	7.7
N279_D280 insE	23.72 ± 1.59	67.5	32.5	2.65 ± 0.07	100.0	0.0	0.0	1.03 ± 0.10	91.9	8.1
Q286_A287 insKK	0.83 ± 0.05	0.0	100.0	0.10 ± 0.00	100.0	0.0	0.0	0 ± 0	0.0	0.0
Q286_A287 insKKAA	0 ± 0	0.0	0.0	0 ± 0	0.0	0.0	0.0	0 ± 0	0.0	0.0
Q286_A287 insKKAAEG	0.64 ± 0.02	12.2	87.8	0.12 ± 0.01	100.0	0.0	0.0	0 ± 0	0.0	0.0

F278_N279 insGP	0 ± 0	0.0	0.0	0.19 ± 0.02	100.0	0.0	0.0	0 ± 0	0.0	0.0
F278_N279 insGPRL	17.28 ± 0.23	45.3	54.7	0.10 ± 0.01	100.0	0.0	0.0	0.85 ± 0.01	93.2	6.8
N279_D280 insP	13.437 ± 0.63	38.1	61.9	1.74 ± 0.04	91.2	3.6	5.3	0.12 ± 0.02	84.1	15.9
Q286_A287 insEM	0 ± 0	0.0	0.0	0.85 ± 0.04	93.5	2.7	3.8	0 ± 0	0.0	0.0
Q286_A287 insEMKA	9.59 ± 0.10	68.4	31.6	0.16 ± 0.02	100.0	0.0	0.0	0.04 ± 0.00	82.8	17.2
Q286_A287 insEMKAEG	1.81 ± 0.14	8.2	91.8	0.13 ± 0.01	100.0	0.0	0.0	0.03 ± 0.02	75.7	24.3
Q286_A287 insEMKAEGK	0 ± 0	0.0	0.0	0.03 ± 0.00	100.0	0.0	0.0	0.17 ± 0.01	84.8	15.2

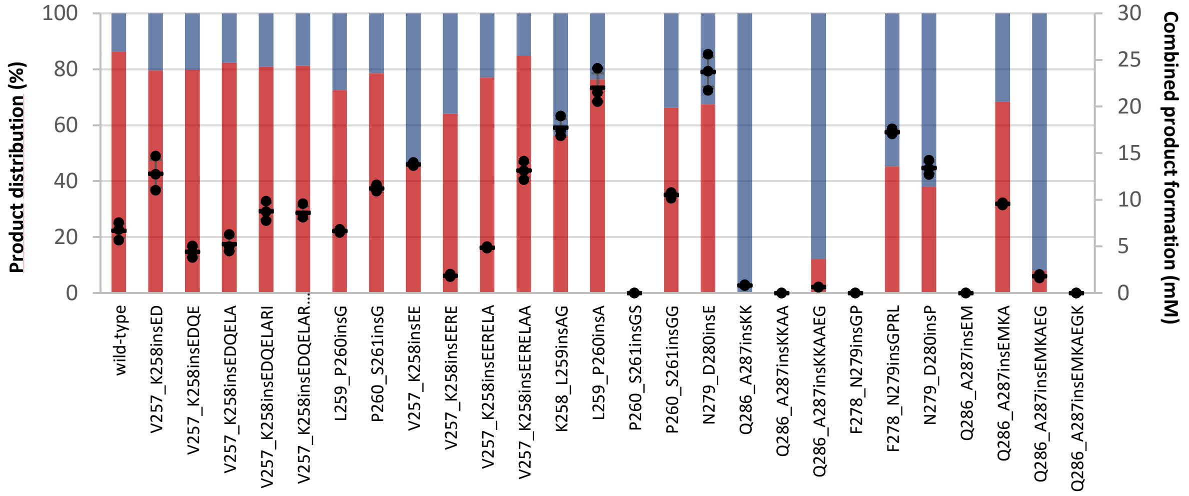

Supplementary Fig. 8: Product distribution and formation of the two products 3-vinylcyclohexa-3,5-diene-1,2-diol 1a (VCHDD, red, bottom bars) and phenylethan-1,2-diol 1b (PED, blue, top bars) for the biotransformation of styrene with the wild-type and insertion variants based on loops in oxygenases from *Phenylobacterium immobile* E. The reactions were performed in technical triplicates (black dots) with average values (horizontal bar) and standard deviations (calculated using Excel version 2016) indicated. Source data are provided as a Source Data file.

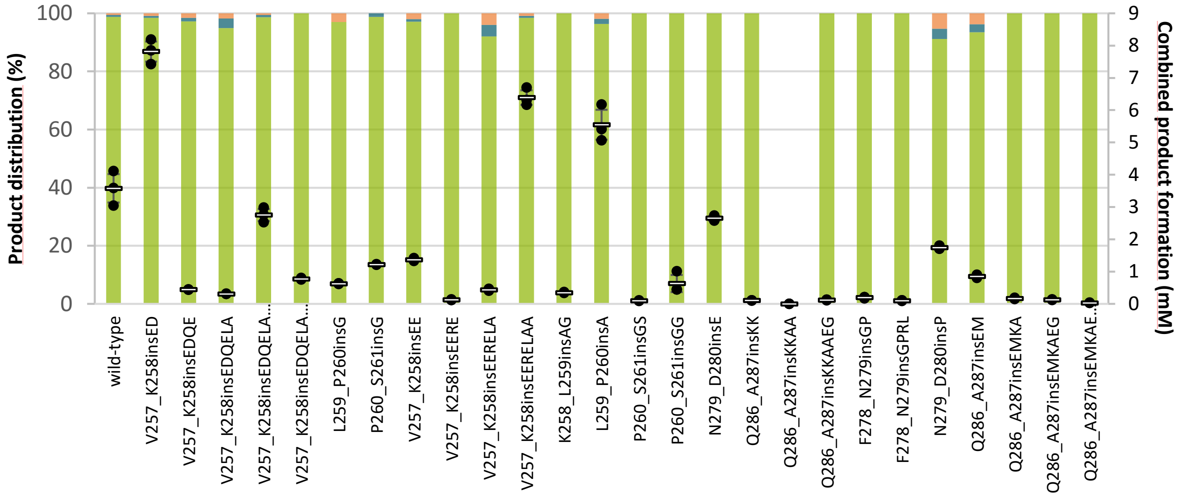

Supplementary Fig. 9: Product distribution and formation of the products (+)-carveol 2a (green, bottom bars), (+)-mentha-1.8-dien-10-ol 2b (MDEO, blue, middle bars) and (+)-perillyl alcohol 2c (orange, top bars) for the biotransformation of (*R*)-limonene 2 with the wild-type and insertion variants based on loops in oxygenases from *Phenylobacterium immobile* E. The reactions were performed in technical triplicates (black dots) with average values (horizontal bar) and standard deviations (calculated using Excel version 2016) indicated. Source data are provided as a Source Data file.

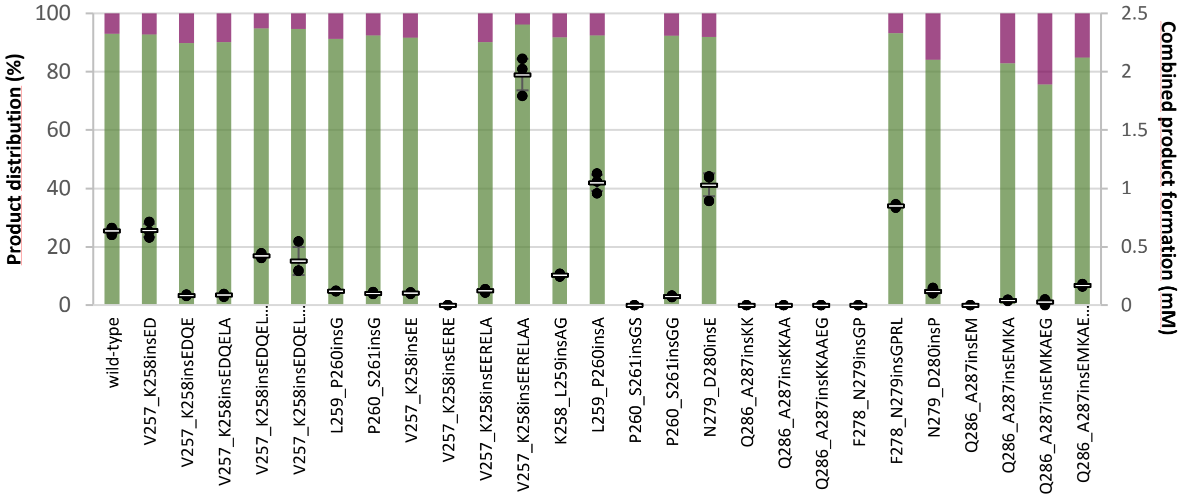

Supplementary Fig. 10: Product distribution and formation of the products 1,2-dihydroxy-3-(2'-pyridyl)cyclohexa-3,5-diene 3a (green, bottom bars) and 2-phenylpyridine-5-ol 3b (purple, top bars) for the biotransformation of 2-phenylpyridine 3 with the wild-type and insertion variants based on loops in oxygenases from in *Phenylobacterium immobile* E. The reactions were performed in technical triplicates (black dots) with average values (horizontal bar) and standard deviations (calculated using Excel version 2016) indicated. Source data are provided as a Source Data file.

Supplementary Table 5: Conversions of styrene 1, (*R*)-limonene 2 and 2-phenylpydridine 3 with the wild-type and deletion variants. Biotransformations were performed in technical triplicates and standard deviations (calculated using Excel version 2016) are indicated. Pf: combined product formation in mM. Regioselectivity in %. Source data are provided as a Source Data file.

	**Pf (mM)**	**Selectivity (%)**		**Pf (mM)**	**Selectivity (%)**			**Pf (mM)**	**Selectivity (%)**	
		**1a**	**1b**		**2a**	**2b**	**2c**		**3a**	**3b**
Wild-type	6.68 ± 0.77	86.4	13.6	3.58 ± 0.44	98.8	0.7	0.5	0.64 ± 0.03	93.0	7.0
E251del	13.14 ± 2.74	84.5	15.5	3.38 ± 0.21	97.2	1.3	1.5	2.18± 0.21	94.7	5.3
P250_M252 del	12.57 ± 2.20	82.4	17.6	2.01 ± 0.16	96.3	1.6	2.1	2.74 ± 0.10	94.2	5.8
L248_L254 del	0 ± 0	0.0	0.0	0.17 ± 0.00	100.0	0.0	0.0	0 ± 0	0.0	0.0
V257del	13.36 ± 0.54	77.1	22.9	2.57 ± 0.16	96.6	1.2	2.1	2.49 ± 0.049	93.6	6.4
Q256_K258 del	15.26 ± 0.56	44.1	55.9	3.68 ± 0.16	97.4	1.1	1.6	1.96 ± 0.04	91.5	8.5
L254_P260 del	0 ± 0	0.0	0.0	0.04 ± 0.00	100.0	0.0	0.0	0 ± 0	0.0	0.0
A283del	15.82 ± 1.99	89.2	10.8	8.92 ± 0.80	98.2	0.8	1.0	10.16 ± 0.47	98.7	1.3
F282_L284 del	19.93 ± 1.97	6.3	93.7	0.60 ± 0.02	100.0	0.0	0.0	3.68 ± 0.11	93.0	7.0
D280_Q286 del	0 ± 0	0.0	0.0	0 ± 0	0.0	0.0	0.0	0 ± 0	0.0	0.0

Supplementary Table 6: Conversions of styrene 1, (*R*)-limonene 2 and 2-phenylpydridine 3 with the wild-type and insertion variants after V257 based on LILI. Biotransformations were performed in technical triplicates and standard deviations (calculated using Excel version 2016) are indicated. Pf: combined product formation in mM. Regioselectivity in %. Source data are provided as a Source Data file.

	Pf (mM)	Selectivity (%)		Pf (mM)	Selectivity (%)			Pf (mM)	Selectivity (%)	
		1a	1b		2a	2b	2c		3a	3b
Wild-type	6.68 ± 0.77	86.4	13.6	3.58 ± 0.44	98.8	0.7	0.5	0.64 ± 0.03	93.0	7.0
V257_K258 insGG	0 ± 0	0.0	0.0	1.96 ± 0.11	97.6	1.2	1.2	0.61 ± 0.00	92.2	7.8
V257_K258 insGGG	0.07 ± 0.01	100.0	0.0	1.49 ± 0.11	98.8	1.2	0.0	0.48 ± 0.01	92.1	7.9
V257_K258 insGGGG	0.22 ± 0.07	100.0	0.0	1.61 ± 0.14	98.9	1.1	0.0	0.52 ± 0.02	92.5	7.5
V257_K258 insGGGGG	0.49 ± 0.22	94.8	5.2	1.32 ± 0.08	98.6	1.4	0.0	0.51 ± 0.01	92.1	7.9
V257_K258 insGGGGGG	0.17 ± 0.09	100.0	0.0	1.32 ± 0.08	99.8	0.2	0.0	0.37 ± 0.03	92.8	7.2
V257_K258 insGS	0.05 ± 0.02	100.0	0.0	2.22 ± 0.04	98.8	1.2	0.0	0.77 ± 0.05	92.6	7.4
V257_K258 insGSG	0.22 ± 0.10	95.6	4.4	1.19 ± 0.27	98.8	1.2	0.0	0.41 ± 0.01	91.6	8.4
V257_K258 insGSGS	0.09 ± 0.03	100.0	0.0	1.13 ± 0.01	100.0	0.0	0.0	0.43 ± 0.02	92.7	7.3
V257_K258 insGSGSG	0.87 ± 0.11	94.1	5.9	1.75 ± 0.47	99.4	0.6	0.0	0.58 ± 0.05	93.1	6.9
V257_K258 insGSGSGS	0.41 ± 0.29	11.3	88.7	1.126 ± 0.16	100.0	0.0	0.0	0.52 ± 0.03	92.5	7.5
V257_K258 insPA	0.03 ± 0.04	100.0	0.0	0.95 ± 0.04	100.0	0.0	0.0	0.31 ± 0.03	92.9	7.1
V257_K258 insPAP	0.10 ± 0.02	100.0	0.0	0.33 ± 0.02	100.0	0.0	0.0	0.08 ± 0.05	97.0	3.0
V257_K258 insPAPA	0 ± 0	0.0	0.0	0.88 ± 0.07	100.0	0.0	0.0	0.27 ± 0.02	93.7	6.3
V257_K258 insPAPAP	0 ± 0	0.0	0.0	0.73 ± 0.01	100.0	0.0	0.0	0.31 ± 0.04	94.9	5.1
V257_K258 insPAPAPA	0.01 ± 0.02	100.0	0.0	0.55 ± 0.06	100.0	0.0	0.0	0.22 ± 0.02	93.5	6.5
V257_K258 insGP	0 ± 0	0.0	0.0	0.58 ± 0.04	100.0	0.0	0.0	0.25 ± 0.05	93.2	6.8
V257_K258 insGPG	0.10 ± 0.04	100.0	0.0	0.84 ± 0.18	100.0	0.0	0.0	0.24 ± 0.01	92.5	7.5
V257_K258 insGPGP	0 ± 0	0.0	0.0	0.58 ± 0.04	100.0	0.0	0.0	0 ± 0	0.0	0.0
V257_K258 insGPGPG	0.07 ± 0.02	100.0	0.0	0.67 ± 0.07	100.0	0.0	0.0	0.27 ± 0.01	93.1	6.9
V257_K258 insGPGPGP	0.09 ± 0.02	100.0	0.0	1.15 ± 0.20	100.0	0.0	0.0	0.17 ± 0.02	94.4	5.6

Supplementary Table 7: Conversions of styrene 1, (*R*)-limonene 2 and 2-phenylpydridine 3 with the wild-type and insertion variants after F278 based on LILI. Biotransformations were performed in technical triplicates and standard deviations (calculated using Excel version 2016) are indicated. Pf: combined product formation in mM. Regioselectivity in %. Source data are provided as a Source Data file.

	Pf (mM)	Selectivity (%)		Pf (mM)	Selectivity (%)			Pf (mM)	Selectivity (%)	
		1a	1b		2a	2b	2c		3a	3b
Wild-type	6.68 ± 0.77	86.4	13.6	3.58 ± 0.44	98.8	0.7	0.5	0.64 ± 0.03	93.0	7.0
F278_N279 insGG	15.74 ± 1.85	50.2	49.8	3.73 ± 0.54	92.7	4.9	2.4	3.49 ± 0.16	94.1	5.9
F278_N279 insGGG	13.44 ± 1.33	55.0	45.0	4.18 ± 0.37	93.3	2.7	4.0	2.48 ± 0.11	93.5	6.5
F278_N279 insGGGG	7.07 ± 1.56	40.4	59.6	3.87 ± 0.30	95.2	1.3	3.5	0.45 ± 0.08	92.3	7.7
F278_N279 insGGGGG	4.59 ± 0.46	55.4	44.6	1.58 ± 0.06	95.5	3.1	1.4	1.37 ± 0.06	92.5	7.5
F278_N279 insGGGGG G	20.69 ± 0.03	49.0	51.0	3.05 ± 0.05	95.7	2.4	2.0	2.31 ± 0.00	92.4	7.6
F278_N279 insGS	14.23 ± 0.67	42.5	57.5	3.45 ± 0.38	94.0	6.0	0.0	0.57 ± 0.04	93.0	7.0
F278_N279 insGSG	13.97 ± 0.33	33.2	66.8	1.60 ± 0.05	91.9	3.2	4.9	0.64 ± 0.04	93.3	6.7
F278_N279 insGSGS	15.41 ± 0.18	34.1	65.9	1.30 ± 0.16	92.5	4.0	3.5	0.39 ± 0.05	91.8	8.2
F278_N279 insGSGSG	20.31 ± 2.89	33.5	66.5	0.82 ± 0.17	94.1	4.4	1.5	0.39 ± 0.08	91.0	9.0
F278_N279 insGSGSGS	15.09 ± 2.03	36.4	63.6	0.90 ± 0.08	95.6	4.4	0.0	0.32 ± 0.04	91.0	9.0
F278_N279 insPA	9.04 ± 1.31	27.8	72.2	0.97 ± 0.04	90.2	6.3	3.6	1.60± 0.32	92.8	7.2
F278_N279 insPAP	11.29 ± 0.61	49.4	50.6	2.21 ± 0.03	92.1	4.6	3.3	2.86 ± 0.16	95.5	4.5
F278_N279 insPAPA	14.23 ± 0.54	52.9	47.1	3.66 ± 0.11	92.7	3.7	3.7	6.16 ± 0.14	96.0	4.0
F278_N279 insPAPAP	14.11 ± 1.25	15.8	84.2	6.03 ± 0.22	92.2	3.4	4.4	1.76 ± 0.01	94.0	6.0
F278_N279 insPAPAPA	9.82 ± 3.56	0.0	100.0	2.29 ± 0.19	92.5	3.5	4.0	0.73 ± 0.00	93.9	6.1
F278_N279 insGP	11.55 ± 0.73	58.2	41.8	1.21 ± 0.03	92.3	4.1	3.7	7.42 ± 0.00	95.6	4.4
F278_N279 insGPG	11.76 ± 1.59	29.4	70.6	6.92 ± 0.68	89.0	5.2	5.7	1.72 ± 0.06	93.5	6.5
F278_N279 insGPGP	13.71 ± 0.08	17.3	82.7	4.44 ± 0.12	87.6	6.8	5.6	1.31 ± 0.04	92.2	7.8
F278_N279 insGPGPG	13.31 ± 0.53	15.4	84.6	3.28 ± 0.19	87.6	3.6	8.8	1.02 ± 0.06	92.5	7.5
F278_N279 insGPGPGP	17.94 ± 1.41	11.6	88.4	2.37 ± 0.23	89.4	3.5	7.1	1.68 ± 0.05	92.2	7.8

Supplementary Table 8: Conversions of styrene 1, (*R*)-limonene 2 and 2-phenylpydridine 3 with the wild-type and insertion variants after Q286 based on LILI. Biotransformations were performed in technical triplicates and standard deviations (calculated using Excel version 2016) are indicated. Pf: combined product formation in mM. Regioselectivity in %. Source data are provided as a Source Data file.

	Pf (mM)	Selectivity (%) 1a	1b	Pf (mM)	Selectivity (%) 2a	2b	2c	Pf (mM)	Selectivity (%) 3a	3b
Wild-type	6.68 ± 0.77	86.4	13.6	3.58 ± 0.44	98.8	0.7	0.5	0.64 ± 0.03	93.0	7.0
Q286_A287 insGG	6.61 ± 0.70	11.2	88.8	0.45 ± 0.04	93.5	1.9	4.6	0.89 ± 0.02	83.6	16.4
Q286_A287 insGGG	8.74 ± 0.97	9.1	90.9	0.57 ± 0.05	91.2	3.6	5.2	1.38 ± 0.10	83.9	16.1
Q286_A287 insGGGG	0 ± 0	0.0	0.0	0 ± 0	0.0	0.0	0.0	0 ± 0	0.0	0.0
Q286_A287 insGGGGG	6.84 ± 1.08	6.2	93.8	0.23 ± 0.01	100.0	0.0	0.0	0.99 ± 0.03	85.4	14.6
Q286_A287 insGGGGGG	6.75 ± 0.26	7.1	92.9	0.19 ± 0.01	100.0	0.0	0.0	0.67 ± 0.04	86.1	13.9
Q286_A287 insGS	6.65 ± 2.69	0.0	100.0	0.48 ± 0.01	96.8	3.2	0.0	0.30 ± 0.01	84.9	15.1
Q286_A287 insGSG	15.00 ± 0.38	0.0	100.0	0.56 ± 0.05	100.0	0.0	0.0	0.36 ± 0.01	80.9	19.1
Q286_A287 insGSGS	0 ± 0	0.0	0.0	0 ± 0	0.0	0.0	0.0	0 ± 0	0.0	0.0
Q286_A287 insGSGSG	17.07 ± 1.03	0.0	100.0	0.25 ± 0.02	100.0	0.0	0.0	0.34 ± 0.01	83.4	16.6
Q286_A287 insGSGSGS	0 ± 0	0.0	0.0	0 ± 0	0.0	0.0	0.0	0 ± 0	0.0	0.0
Q286_A287 insPA	16.32 ± 1.13	30.2	69.8	1.87 ± 0.22	89.0	5.5	5.5	1.26 ± 0.08	86.4	13.6
Q286_A287 insPAP	19.14 ± 1.6	15.6	84.4	1.35 ± 0.08	89.1	5.4	5.5	1.34 ± 0.07	87.8	12.2
Q286_A287 insPAPA	15.10 ± 0.94	7.5	92.5	0.96 ± 0.04	89.5	4.2	6.3	1.01 ± 0.01	85.8	14.2
Q286_A287 insPAPAP	2.39 ± 0.50	12.2	87.8	0.88 ± 0.05	92.4	5.1	2.5	0.78 ± 0.03	91.2	8.8
Q286_A287 insPAPAPA	18.93 ± 1.19	7.1	92.9	0.73 ± 0.078	93.2	4.9	1.9	0.96 ± 0.02	89.6	10.4
Q286_A287 insGP	11.52 ± 0.79	11.8	88.2	0.66 ± 0.05	92.4	3.4	4.2	1.37 ± 0.07	86.0	14.0
Q286_A287 insGPG	13.35 ± 1.26	7.5	92.5	1.23 ± 0.08	95.6	2.4	2.0	1.86 ± 0.03	79.8	20.2
Q286_A287 insGPGP	13.94 ± 0.61	7.9	92.1	0.59 ± 0.05	91.1	3.7	5.1	1.23 ± 0.05	88.5	11.5
Q286_A287 insGPGPG	14.52± 0.61	5.8	94.2	0.86 ± 0.04	92.7	3.8	3.4	1.92 ± 0.08	81.4	18.6
Q286_A287 insGPGPGP	15.67 ± 0.24	6.5	93.5	0.42 ± 0.03	88.1	7.0	4.8	1.04 ± 0.01	89.5	10.5

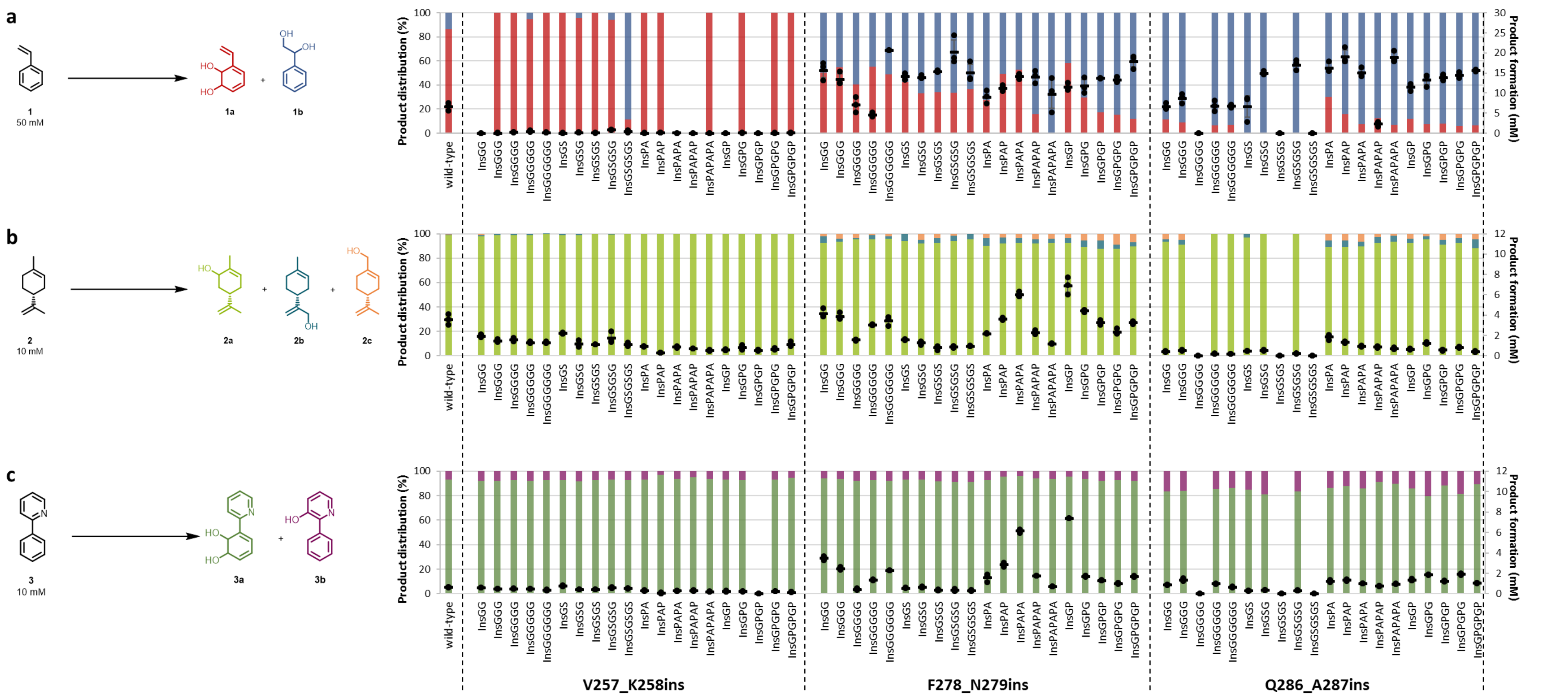

Supplementary Fig. 11: Biotransformation results with variants derived from the LILI approach. a, Product formation and distribution of the products **1a** (red, bottom bars) and **1b** (blue, top bars) obtained during the biotransformation of **1** with the wild-type and variants of the LILI library. **b**, Product formation and distribution of the products **2a** (green, bottom bars), **2b** (blue, middle bars) and **2c** (orange, top bars) obtained during the biotransformation of **2** with the wild-type and variants of the LILI library. **c**, Product formation and distribution of the products **3a** (green, bottom bars) and **3b** (purple, top bars) obtained during the biotransformation of **3** with the wild-type and variants of the LILI library. The reaction conditions as mentioned in Fig. 2 were applied, the substrate concentrations are indicated in the reaction equation. The reactions were performed in technical triplicates (black dots) with average values (horizontal bar) and standard deviations (calculated using Excel version 2016) indicated. Error bars may be covered by markers. Source data are provided as a Source Data file.

Supplementary Table 9: Different wild-types and active-site variants known from literature compared to selected loop variants from this study. t.w.: this work. n.d.: not determined. Source data are provided as a Source Data file.

Substrate	Group	Variant	Product formation (%)	Selectivity (%) 1a	1b		Annotation	Source
**Styrene 1**	**CDO wild-type**		13.4 ± 1.55	86.4	13.6 (*ee* 18.4 ± 1.1 *R*-**1b**)		50 mM substrate 0.1 g_{cww}/ml *E. coli*	t.w.
**Styrene 1**	**Literature**	**CDO wild-type (*Pseudomonas fluorescens* IP01)**	71 ± 6	99.7	0.3 (*ee* 43 ± 3 *R*-**1b**)		10 mM substrate, 0.2 g_{cww}/ml *E. coli*	[1]
**Styrene 1**	**Literature**	**NDO wild-type (*Pseudomonas sp.* NCIB 9816-4)**	74 ± 3	0	>99 (*ee* 78 ± 1 *R*-**1b**)		10 mM substrate, 0.2 g_{cww}/ml *E. coli*	[2]
**Styrene 1**	**Literature**	**TDO wild-type (*Pseudomonas. putida* F1)**	9	85	15 (*ee* >99 *R*-**1b**)		10 mM substrate	[3]
**Styrene 1**	**Active-site**	**CDO_M232A**	97 ± 10	8	92 (*ee* 95 ± 1 *R*-**1b**)		10 mM substrate, 0.2 g_{cww}/ml *E. coli*	[1]
**Styrene 1**	**Active-site**	**NDO_H295A**	92 ± 2	<1	>99 (*ee* 79 ± 1 *R*-**1b**)		10 mM substrate, 0.2 g_{cww}/ml *E. coli*	[2]
**Styrene 1**	**Active-site**	**TDO_T365N**	14	24	76 (*ee* >99 *R*-**1b**)		10 mM substrate,	[3]
**Styrene 1**	**Loop**	**CDO_N279_D280insE**	47.4 ± 3.2	67.5	32.5 (*ee* 11.4 ± 5.8 *R*-**1b**)		50 mM substrate 0.1 g_{cww}/ml *E. coli*	t.w.
**Styrene 1**	**Loop**	**CDO_F282A**	24.6 ± 5.7	95.0	5.0 (*ee* 72.7 ± 0.6 *R*-**1b**)		50 mM substrate 0.1 g_{cww}/ml *E. coli*	t.w.
**Styrene 1**	**Loop**	**CDO_Q286_A287insGSGSG**	34.1 ± 2.1	<1	>99 (*ee* 6.4 ± 0.2 *R*-**1b**)		50 mM substrate 0.1 g_{cww}/ml *E. coli*	t.w.
**Styrene 1**	**Loop**	**CDO_F282_L284del**	39.9 ± 4.0	6.3	93.7 (*ee* 1.4 ± 0.6 *R*-**1b**)		50 mM substrate 0.1 g_{cww}/ml *E. coli*	t.w.
				**2a**	**2b**	**2c**		
**(*R*)-Limonene 2**	**CDO wild-type**		35.8 ± 4.4	98.8 (*ee* 99.6 ± 0.1 1*R*,5*S*-**2a**)	0.7	0.5	0.1 g_{cww}/ml *E. coli*	t.w.
**(*R*)-Limonene 2**	**Literature**	**CDO wild-type (*Pseudomonas fluorescens* IP01)**	46 ± 10	> 95 (*ee* > 98 ± 0.1 1*R*,5*S*-**2a**)	<5	0	0.2 g_{cww}/ml *E. coli*	[1]
**(*R*)-Limonene 2**	**Literature**	**CDO wild-type (*Pseudomonas putida S1*)**	40	>95 (*ee* >98 1*R*,5*S*-**2a**)	0	0	OD_{600} = 3.0 6.2 mM substrate	[4]
**(*R*)-Limonene 2**	**Literature**	**NDO wild-type (*Pseudomonas sp.* NCIB 9816-4)**	n.d.	92	8	0	0.2 g_{cww}/ml *E. coli*	[2]
**(*R*)-Limonene 2**	**Active-site**	**CDO_M232A**	>99	95 (*ee* > 98 1*R*,5*S*-**2a**)	5	0	0.2 g_{cww}/ml *E. coli*	[1]
**(*R*)-Limonene 2**	**Active-site**	**NDO_H295A_V260A**	213% of wild-type	93 (*ee* 62 1*R*,5*S*-**2a**)	7	0	0.2 g_{cww}/ml *E. coli*	[2]
**(*R*)-Limonene 2**	**Loop**	**CDO_A283del**	89.2 ± 8.0	98.2 (*ee* 62 1*R*,5*S*-**2a**)	0.8	1.0	0.1 g_{cww}/ml *E. coli*	t.w.
**(*R*)-Limonene 2**	**Loop**	**CDO_L284G**	20.8 ± 1.9	30.8 (*ee* >99 1*R*,5*S*-**2a**)	19.4	49.7	0.1 g_{cww}/ml *E. coli*	t.w.
**(*R*)-Limonene 2**	**Loop**	**CDO_M289A**	19.4 ± 0.9	54.1 (*ee* >99 1*R*,5*S*-**2a**)	6.2	39.8	0.1 g_{cww}/ml *E. coli*	t.w.

				3a	3b		
2-Phenyl-pyridine 3	**CDO wild-type**		6.4 ± 0.3	93.0 (*ee* >99 1*S*, 2*R*-**3a**)	7.0		t.w.
	**Literature**	**TDO wild-type** (***Pseudomonas putida*** **UV4)**	1% yield	100 (*ee* >98 1*S*, 2*R*-**3a**)			5
		**NDO wild-type** (***Pseudomonas*** **sp. NCIB 9816-4)**	25% yield	≥95		≤ 5% *cis*-3,4-dihydrodiol (*ee* >98 1*S*, 2*R*) was formed	5
		**BPDO wild-type (*Sphingomomas yanoikuyae*** **(B8/36))**	59% yield	≥95			5
	**Loop**	**CDO_A283del**	>99	98.7 (*ee* >99 1*S*, 2*R*-**3a**)	1.3		t.w.
		**CDO_F282A**	99.9 ± 4.0	98.6 (*ee* >99 1*S*, 2*R*-**3a**)	1.4		t.w.
		**CDO_Q286_A287insGPG**	18.6 ± 0.3	79.8 (*ee* >99 1*S*, 2*R*-**3a**)	20.2		t.w.

Supplementary Table 10: Enantiomeric excesses of the biotransformations of styrene 1, (*R*)-limonene 2 and 2-phenylpydridine 3 with the selected variants from Supplementary Table S19, and saturation and deletion variants. Biotransformations were performed in technical triplicates and standard deviations (calculated using Excel version 2016) are indicated. Enantiomeric excess in %. n.d.: not determined. -: not detected. Source data are provided as a Source Data file.

	**Product (%)**		
**Variant**	**Phenylethan-1,2-diol**<br><br>**1b**	**Carveol**<br><br>**2a**	**1,2-Dihydroxy-3-(2'pyridyl)-cyclohexa-3,5-diene**<br>**3a**
Wild-type	18.43 ± 1.08 (*R*)-**1b**	99.73 ± 0.13 (1*R*,5*S*)-**2a**	>99 % (+)-(1*S*,2*R*)-**3a**
F282A	72.72 ± 0.63 (*R*)-**1b**	n.d.	>99 % (+)-(1*S*,2*R*)-**3a**
M289A	n.d.	99.97 ± 0.05 (1*R*,5*S*)-**2a**	>99 % (+)-(1*S*,2*R*)-**3a**
N279_D280insE	11.42 ± 5.84 (*R*)-**1b**	n.d.	>99 % (+)-(1*S*,2*R*)-**3a**
A283del	42.00 ± 0.59 (*R*)-**1b**	>99.9 (1*R*,5*S*)-**2a**	>99 % (+)-(1*S*,2*R*)-**3a**
F282_L284del	1.49 ± 0.15 (*R*)-**1b**	93.30 ± 0.72 (1*R*,5*S*)-**2a**	>99 % (+)-(1*S*,2*R*)-**3a**
F278V	41.32 ± 6.64 (*R*)-**1b**	99.99 ± 0.01 (1*R*,5*S*)-**2a**	>99 % (+)-(1*S*,2*R*)-**3a**
F282V	69.83 ± 3.61 (*R*)-**1b**	>99.9 (1*R*,5*S*)-**2a**	>99 % (+)-(1*S*,2*R*)-**3a**
F282T	-	>99.9 (1*R*,5*S*)-**2a**	>99 % (+)-(1*S*,2*R*)-**3a**
L284G	26.03 ± 1.08 (*R*)-**1b**	>99.9 (1*R*,5*S*)-**2a**	>99 % (+)-(1*S*,2*R*)-**3a**
Q286F	15.83 ± 0.23 (*R*)-**1b**	>99.9 (1*R*,5*S*)-**2a**	>99 % (+)-(1*S*,2*R*)-**3a**
I288S	15.56 ± 0.70 (*S*)-**1b**	99.76 ± 0.02 (1*R*,5*S*)-**2a**	>99 % (+)-(1*S*,2*R*)-**3a**
I288T	1.17 ± 0.83 (*S*)-**1b**	>99.9 (1*R*,5*S*)-**2a**	>99 % (+)-(1*S*,2*R*)-**3a**
M289K	23.97 ± 0.44 (*R*)-**1b**	>99.9 (1*R*,5*S*)-**2a**	>99 % (+)-(1*S*,2*R*)-**3a**

The enantiomeric excess of **3a** was determined by comparison to two in-house standards of (+)-(1*S*,2*R*)-**3a**. The configuration was determined by optical rotation.[5] Multiple columns and methods were applied, which all reveal only one enantiomer.

Supplementary Table 11: Diastereomeric excesses of the biotransformations (*R*)-limonene 2 with the selected variants from Supplementary Supplementary Table 10, saturation and deletion variants. Biotransformations were performed in technical triplicates and standard deviations (calculated using Excel version 2016) are indicated. Diastereomeric excess in %. Source data are provided as a Source Data file.

**Variant**	**Carveol 2a**
Wild-type	94.70 ± 0.37 (1*R*,5*S*)-**2a**
M289A	97.05 ± 0.26 (1*R*,5*S*)-**2a**
A283del	87.27 ± 1.14 (1*R*,5*S*)-**2a**
F282_L284del	98.41 ± 0.02 (1*R*,5*S*)-**2a**
F278V	55.10 ± 2.53 (1*R*,5*S*)-**2a**
F282V	99.14 ± 0.01 (1*R*,5*S*)-**2a**
F282T	99.41 ± 0.20 (1*R*,5*S*)-**2a**
L284G	38.14 ± 1.81 (1*R*,5*S*)-**2a**
Q286F	98.11 ± 0.08 (1*R*,5*S*)-**2a**
I288S	90.23 ± 1.53 (1*R*,5*S*)-**2a**
I288T	99.12 ± 0.06 (1*R*,5*S*)-**2a**
M289K	88.02 ± 0.82 (1*R*,5*S*)-**2a**

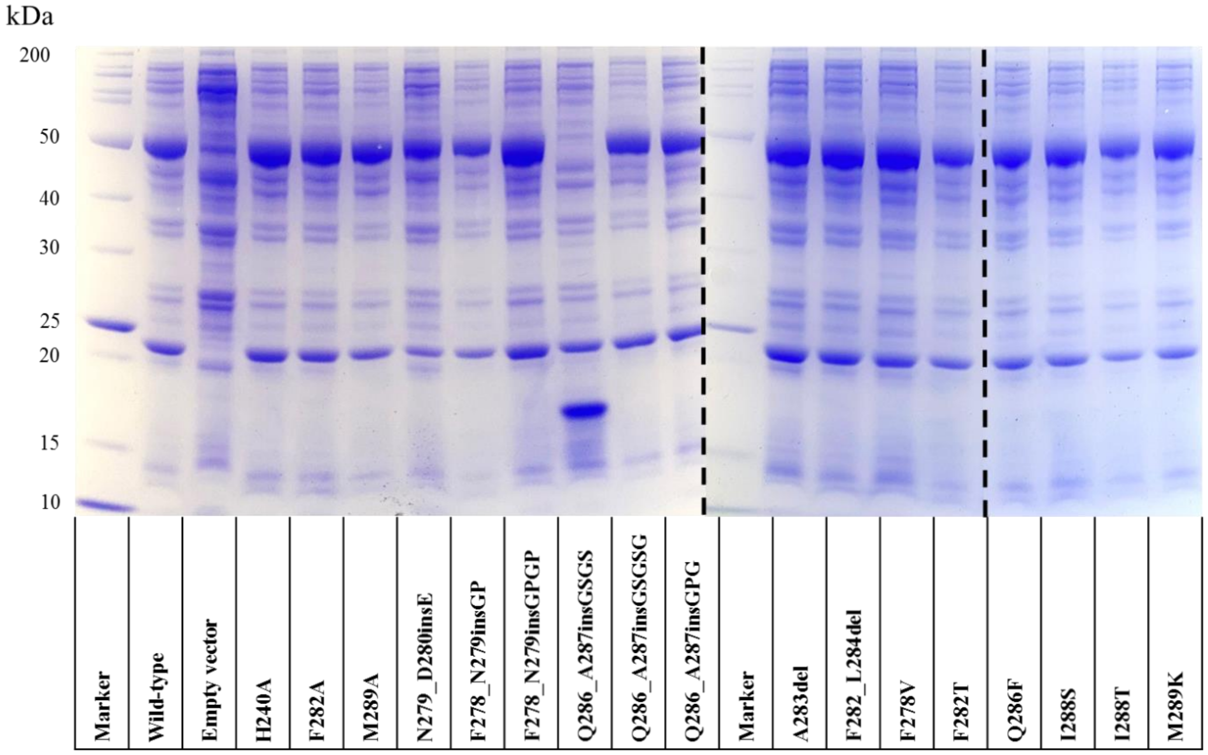

Supplementary Fig. 12: SDS-PAGE analysis of the empty vector, expressed wild-type and selected variants of the CDO. The analysis of the expression levels was performed in a RunBlue 12% Bis-Tris Gel from Expedeon (Abcam, Berlin, Germany) with PageRuler™ Unstained Protein Ladder (Thermo Scientific, Waltham, USA) as marker. Whole cell samples were standardized to OD_{600} = 4. The bands of the α-subunit (52 kDa) and β-subunit (23 kDa) are clearly visible, except for the α-subunit of Q286_A287insGSGS. The bands of the reductase (44 kDa) and ferredoxin (12 kDa) were not observed. Source data are provided as a Source Data file. Dashed lines represent different gels and vertically sliced image. SDS-PAGE analysis was performed independently 15 times for the wildtype with comparable results. Other variants were analyzed *via* SDS-PAGE with the wildtype as positive control at least once and repeated when preparation errors occurred.

All obtained variants were analyzed *via* non-native SDS-PAGE of the whole cells as shown in Supplementary Fig. S12. All variants, also these which showed no activity for any of the tested variants, were expressed. No native SDS-PAGE was observed, so no conclusions about the solubility or correct folding could be drawn.

Supplementary Note 1: Synthesis of (+)-mentha-1.8-dien-10-ol (2b)

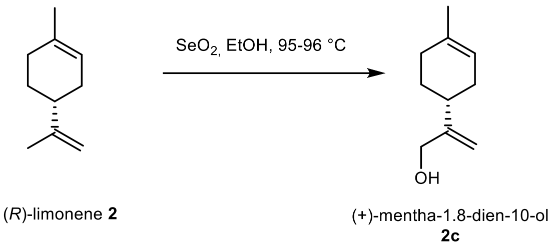

The synthesis of (+)-mentha-1.8-dien-10-ol **2b** was performed according to Thomas and Bucher.[6]

Yellow oil; ^{1}H NMR (500 MHz, $CDCl_3$,): δ 1.43-1.56 (2 H, m), 1.66 (3 H, s), 1.78-2.23 (6 H, m), 4.18 (2 H, d, *J* 5.7), 4.91 (1 H, s), 5.04-5.07 (1 H, m), 5.40 (1 H, br s); ^{13}C NMR (500 MHz $CDCl_3$,): δ 23.48, 28.18, 30.53, 31.36, 36.91, 65.19, 107.83, 120.45, 133.85, 153.67. MS (GC, EI): m/z (%) =153 (1, M^+), 152 (11.8, M), 134 (47), 121 (17), 119 (91), 107 (14), 106 (100), 105 (39), 94 (34), 93 (61), 92 (46), 91 (82), 84 (25), 83 (16), 79 (80), 77 (42), 68 (60), 67 (72), 65 (16)

GC-MS and NMR analysis revealed contaminations with formed perillyl alcohol.

Supplementary Note 2: Determination of product formation, distribution, enantiomeric excess and diastereomeric excess

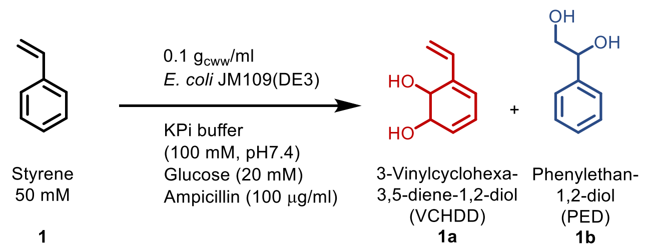

Supplementary Fig. 13: Conversion of styrene to 3-vinylcyclohexa-3,5-diene-1,2-diol 1a and 1-phenylethan-1,2-diol 1b

3-vinylcyclohexa-3,5-diene-1,2-diol (1a)

MS (GC, EI) m/z (%) = 139 (1.7, M+), 138 (19, M), 120 (94), 109 (21), 92 (39), 91 (100), 81 (19), 79 (23), 77 (24), 65 (17)

1-phenyl-1,2-ethanediol (1b)

Rieske non-heme iron oxygenase (RO)-catalyzed biotransformations

MS (GC, EI) m/z (%) = 139 (0.8, M^+), 138 (9, M), 108 (9), 107 (100), 91 (4), 79 (48), 77 (28)

Standard Sigma Aldrich

MS (GC, EI) m/z (%) = 139 (2, M^+), 138 (23, M), 108 (22), 107 (100), 91 (10), 79 (100), 77 (77)

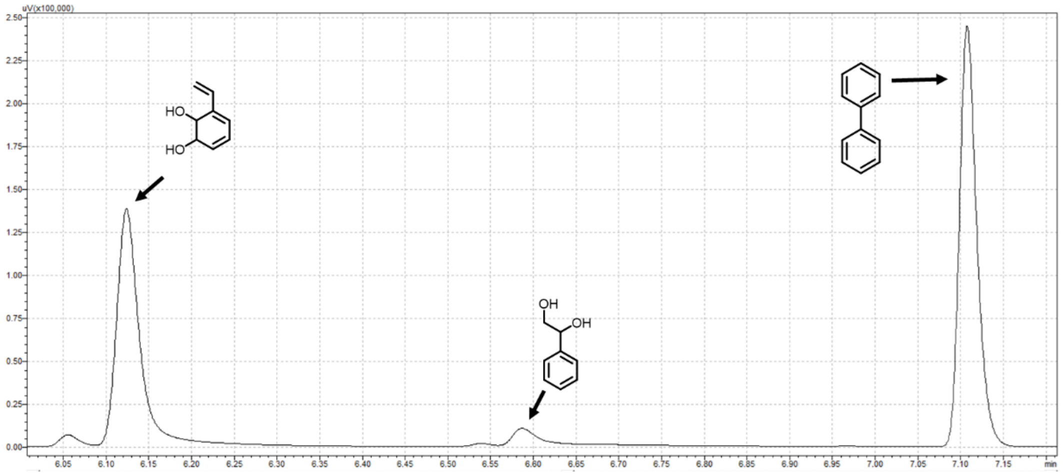

Supplementary Fig. 14: GC-FID chromatogram of CDO wild-type catalyzed biotransformation of styrene 1.

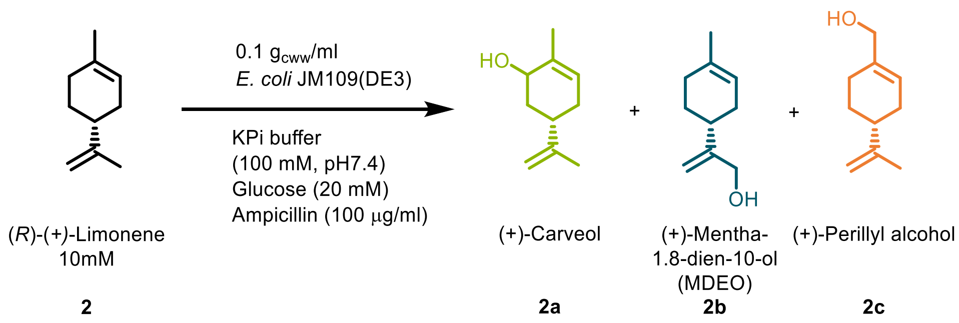

Supplementary Fig. 15: Conversion of (*R*)-(+)-limonene 2 to (+)-carveol 2a, (+)-mentha-1.8-dien-10-ol 2b and (+)-perillyl alcohol 2c.

(+)-carveol 2a

Rieske non-heme iron oxygenase (RO)-catalyzed biotransformation

MS (GC, EI) m/z (%) = 153 (1.2, M^+), 152 (11, M), 137 (11), 119 (11), 109 (100), 108 (13), 95 (13), 94 (4), 93 (11), 92 (5), 91 (15), 84 (47), 83 (25), 81 (10), 80 (8), 79 (10), 69 (17), 55 (18)

Standard Sigma-Aldrich

MS (GC, EI) m/z (%) = 153 (0.3, M+), 152 (3, M), 137 (68), 119 (34), 109 (77), 108 (12), 95 (24), 94 (25), 93 (27), 92 (17), 91 (24), 84 (100), 83 (44), 81 (25), 80 (25), 79 (24), 69 (36), 55 (31)

(+)-mentha-1.8-dien-10-ol 2b

Rieske non-heme iron oxygenase (RO)-catalyzed biotransformation

MS (GC, EI) m/z (%) = 153 (5, M^+), 152 (18, M), 134 (63), 121 (27), 119 (100), 107 (22), 106 (79), 105 (50), 95 (29), 94 (49), 93 (80), 92 (54), 91 (82), 84 (40), 79 (95), 77 (42), 68 (95), 67 (98), 55 (49)

(+)-perillyl alcohol 2c

Rieske non-heme iron oxygenase (RO)-catalyzed biotransformation

MS (GC, EI) m/z (%) = 153 (1.6, M^+), 152 (9, M), 121 (83), 119 (31), 109 (26), 108 (28), 107 (37), 106 (18), 105 (22), 95 (38), 94 (52), 93 (95), 91 (53), 81 (37), 79 (100), 77 (30), 68 (90), 67 (80), 55 (44)

Standard Sigma-Aldrich

MS (GC, EI) m/z (%) = 153 (2, M+), 152 (18, M), 121 (87), 119 (48), 109 (35), 108 (33), 107 (14), 106 (28), 105 (28), 95 (18), 94 (18), 93 (90), 91 (61), 81 (29), 79 (100), 77 (32), 68 (86), 67 (77), 55 (39)

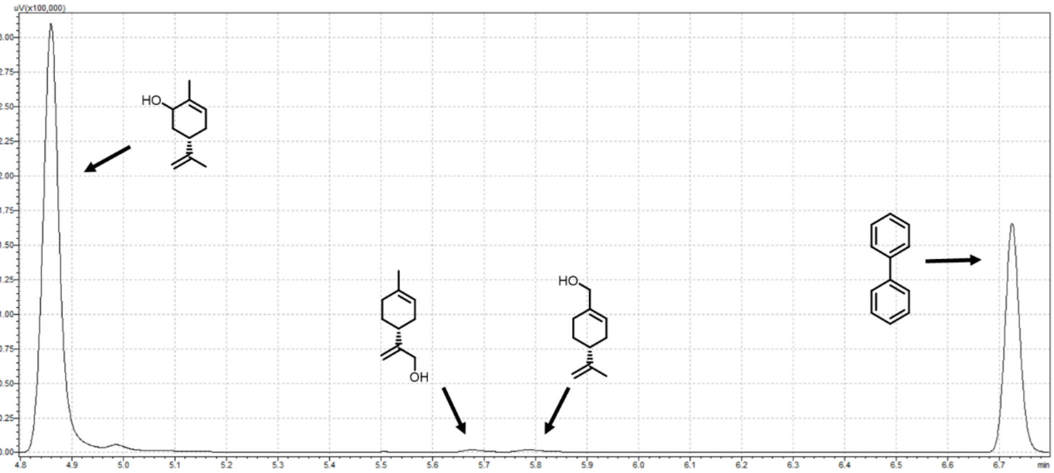

Supplementary Fig. 16: GC-FID chromatogram of CDO wild-type catalyzed biotransformation of (*R*)-(+)-limonene 2.

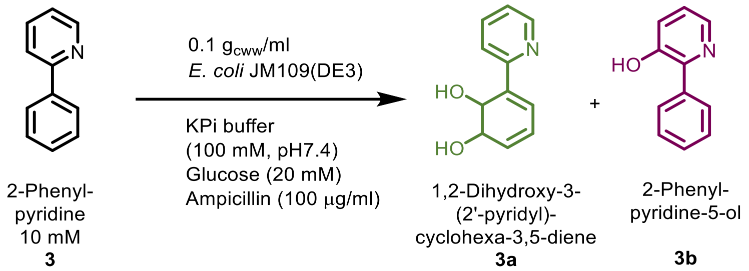

Supplementary Fig. 17: Conversion of 2-phenylpyridine 3 to 1,2-dihydroxy-3-(2'-pyridyl)-cyclohexa-3,5-diene 3a and 2-phenyl-pyridine-5-ol 3b.

1,2-dihydroxy-3-(2'-pyridyl)-cyclohexa-3,5-diene 3a

Rieske non-heme iron oxygenase (RO)-catalyzed biotransformations

MS (LC, ESI, positive) m/z (%) = 191 (M^+, 12), 190 (100, M), 189 (58), 173 (4), 172 (35)

Standard from in-house library, derived from an upscaling of biotransformation of 2-phenylpyridine with TDO Wild-type

MS (LC, ESI, positive) m/z (%) = 191 (M^+, 6), 190 (100, M), 173 (6), 172 (48)

Brown solid; $[\alpha]_D$ +161 (*c* 1.1, MeOH); ^{1}H NMR δ ($CDCl_3$, 500 MHz), 4.50 (1 H, m, H-1), 4.93 (1 H, d, $J_{1,2}$ 9.7, H-2), 6.18 (2 H, m, H-5, H-6), 6.65 (1 H, d, $J_{4,5}$ 5.4, H-4), 6.89 (1 H, dd, $J_{4',3'}$ 9.9, $J_{4',5'}$ 2.5, H-4'), 7.51 (1 H, d, $J_{3',4'}$ 5.4, H-3'), 7.6 (1 H, d, $J_{5',4'}$ 8, H-5'), 8.51 (1 H, d, $J_{6',5'}$ 4.6, H-6'); ^{13}C NMR δ (500 MHz, $CDCl_3$) 67.26, 69.24, 119.98, 122.13, 123.58, 124.82, 131.44, 136.21, 136.97, 148.19, 157.33; [5]

2-phenyl-pyridine-5-ol 3b

Rieske non-heme iron oxygenase (RO)-catalyzed biotransformations

MS (LC, ESI, positive) m/z (%) = 173 (12, M^+), 172 (100, M)

Standard Fluorochem

MS (LC, ESI, positive) m/z (%) = 173 (13, M^+), 172 (100, M)

MS (GC, EI) m/z (%) = 172 (5.6, M^+), 171 (52, M), 170 (100), 169 (2), 144 (1), 143 (7), 142 (7), 141 (3), 140 (2), 117 (6), 116 (9), 115 (27)

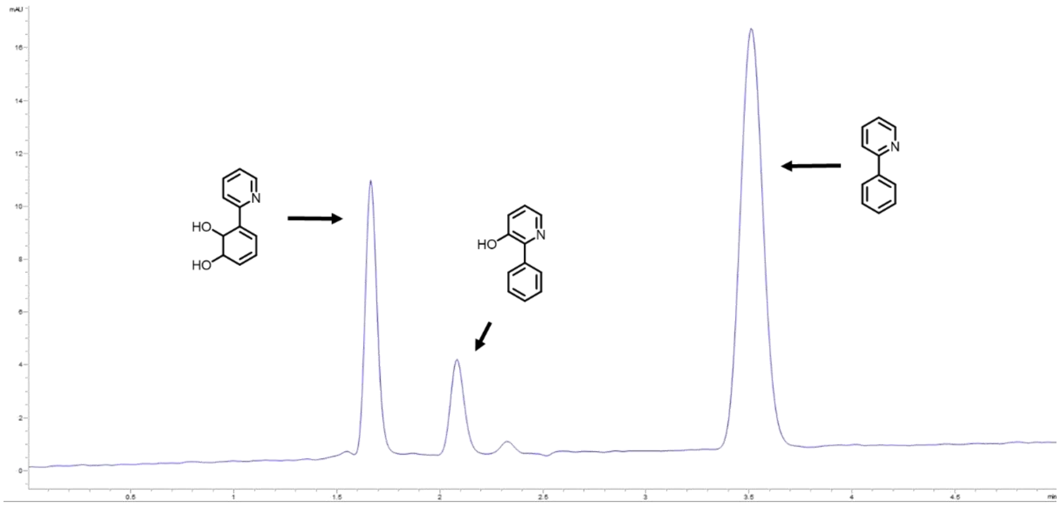

Supplementary Fig. 18: DAD chromatogram of CDO wild-type catalyzed biotransformation of 2-phenylpyridine at a wavelength of 210 nm.

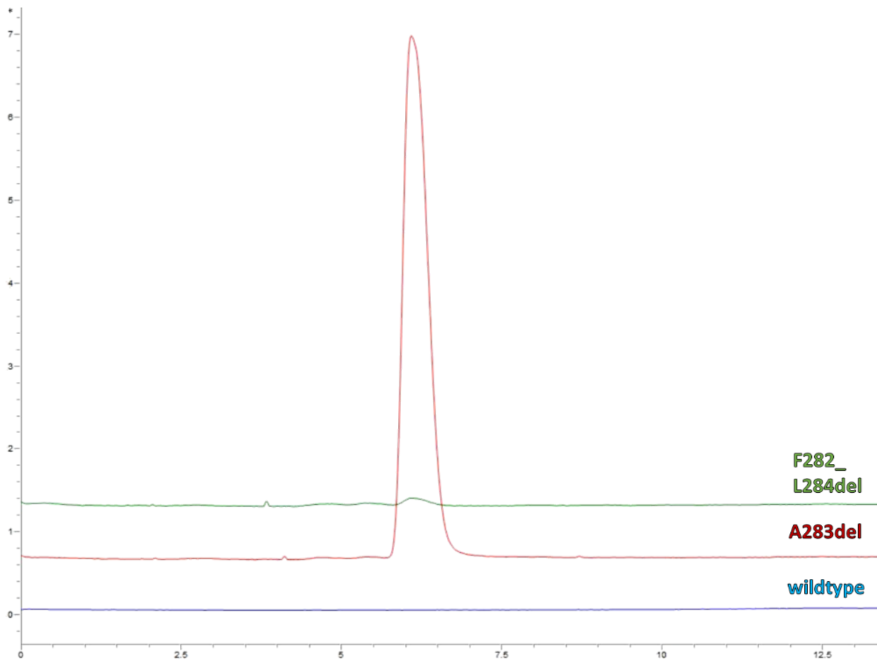

Supplementary Fig. 19: LC-MS spectrum of the biotransformation of phenazone in SIM-mode (m/z 222). Comparison between the wild-type (blue, bottom line) with no product formation, F282_L284del (green, top line) with product traces and A283del (red, middle line) with clear product formation.

Product formations and distribution

The quantification of the different products was performed with biphenyl as internal standard under the same conditions as the corresponding biotransformations. 1 M DMSO stocks of the corresponding products were applied for standard solutions, incubated, extracted and analyzed. LC/MS analysis of the in-house standard of 1,2-dihydroxy-3-(2'-pyridyl)-cyclohexa-3,5-diene (**3a**) showed traces of 2-phenyl-pyridine-5-ol (**3b)**. Based on the corresponding calibration curve, the amount of 2-phenyl-pyridine-5-ol was subtracted from the used concentration of 1,2-dihydroxy-3-(2'-pyridyl)-cyclohexa-3,5-diene standard to ensure correct concentrations.

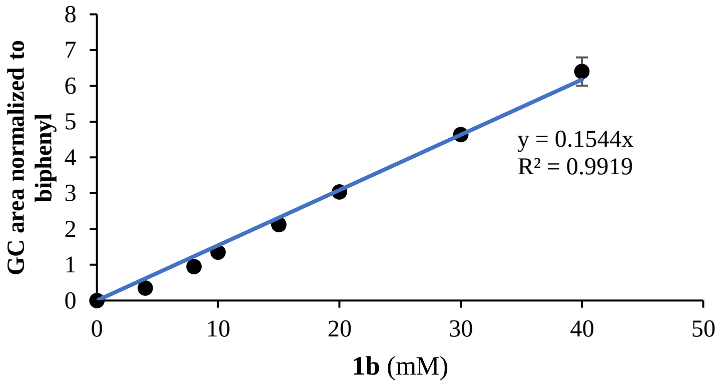

Supplementary Fig. 20. Calibration curve of 1-phenyl-1,2-ethandiol (1b). Data acquisition was performed in technical triplicates per concentration with average values and standard deviations (calculated using Excel version 2016) are indicated. Error bars may be covered by markers. Source data are provided as a Source Data file.

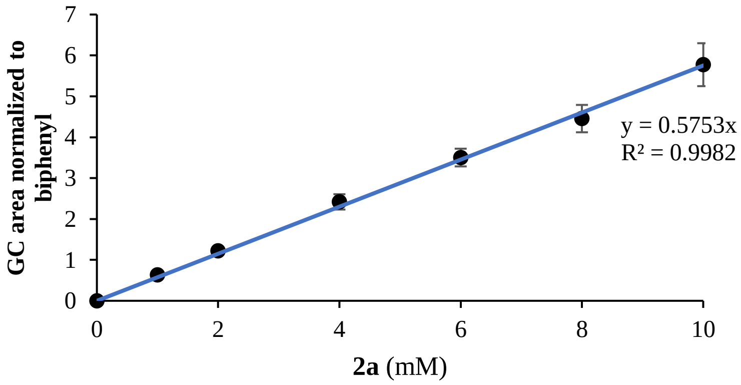

Supplementary Fig. 21. Calibration curve of the mixture of isomers of (-)-carveol (2a). Data acquisition was performed in technical triplicates per concentration with average values and standard deviations (calculated using Excel version 2016) are indicated. Error bars may be covered by markers. Source data are provided as a Source Data file.

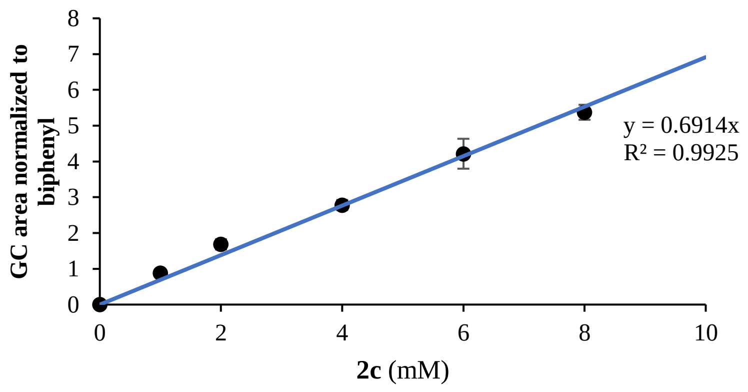

Supplementary Fig. 22. Calibration curve of (+)-perillyl alcohol (2c). Data acquisition was performed in technical triplicates per concentration with average values and standard deviations (calculated using Excel version 2016) are indicated. Error bars may be covered by markers. Source data are provided as a Source Data file.

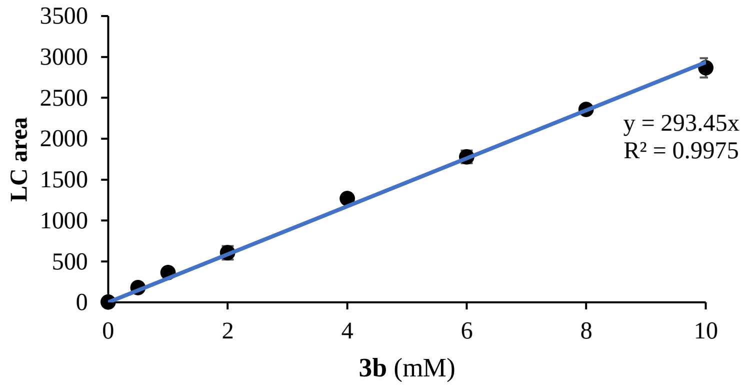

Supplementary Fig. 23. Calibration curve of 2-phenyl-pyridine-5-ol (3b). Data acquisition was performed in technical triplicates per concentration with average values and standard deviations (calculated using Excel version 2016) are indicated. Error bars may be covered by markers. Source data are provided as a Source Data file.

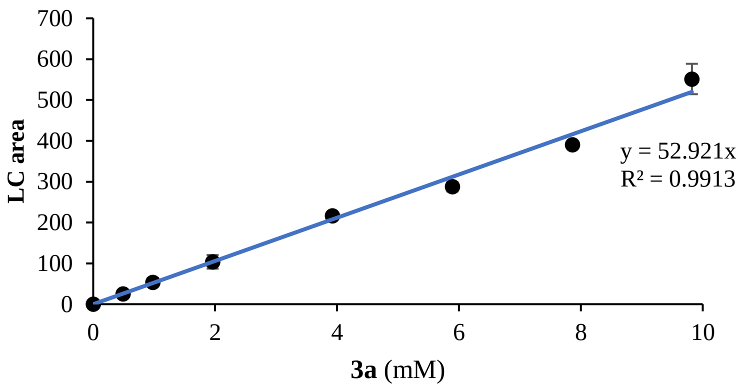

Supplementary Fig. 24. Calibration curve of 1,2-dihydroxy-3-(2’-pyridyl)-cyclohexa-3,5-diene (3a). Data acquisition was performed in technical triplicates per concentration with average values and standard deviations (calculated using Excel version 2016) are indicated. Error bars may be covered by markers. Source data are provided as a Source Data file.

The product 3-vinylcyclohexa-3,5-diene-1,2-diol **1a** polymerizes when concentrated in vacuo, so the synthesis of a standard for quantification is not possible. Also, the synthesis of (+)-mentha-1.8-dien-10-ol **2b** yielded a standard with impurities with perillyl alcohol, despite repeated column chromatography. To ensure correct quantification, we used the relative response factor to determine the concentrations in reference to a standard.[7,8] For **1a**, we used **1b** as standard, while **2c** was applied as reference standard for **2b**. The RF values were calculated from the effective carbon numbers (ECN) of the respective compound (ECN_X) in relation the the ECN of the standard (ECN_{STD}) with equation (1).

$$RF = \frac{ECN_X}{ECN_{STD}} \qquad (1)$$

Calibration curves were determined by the quantification of the analytes by external calibration and normalization by the calculated RF values (Supplementary Table 12).

Supplementary Table 12: Calculated ECN and RF values.

Compound	ECN	RF
1a	6.2	0.919
1b	6.5	
2b	9.3	1
2c	9.3	

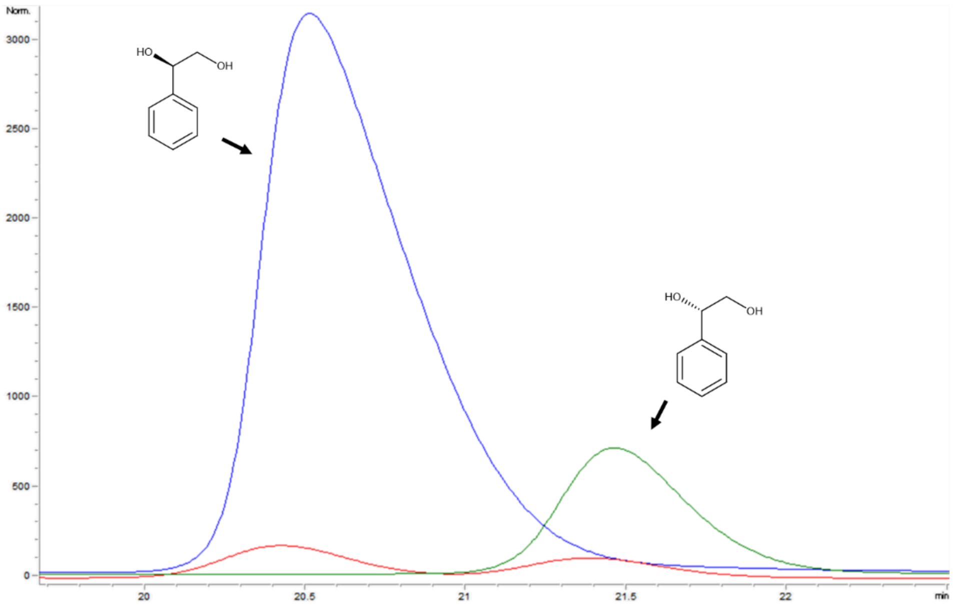

Supplementary Fig. 25: Chiral HPLC analysis of 1-phenyl-1,2-ethandiol 1b enantiomers of the commercially available standards of (*R*)-1b (blue, Sigma Aldrich, 99 % optical purity), (*S*)-1b (green, Sigma Aldrich, 99 % optical purity) and the RO-catalyzed biotransformation of 1 (red).

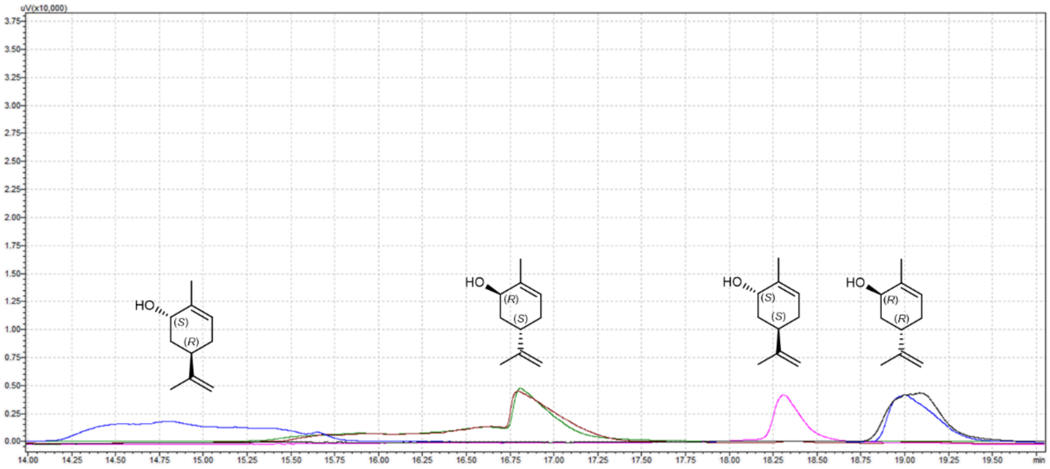

Supplementary Fig. 26: Chiral GC-FID analysis of carveol 2a enantiomers. Commercially available isomer mixture of (-)-carveol (black, Sigma Aldrich, 97 %) containing (1*S*,5*R*)-**2a** and (1*R*,5*R*)-**2a**. (1*S*,5*S*)-carveol (pink) from our in-house library, synthesis according to Bermejo *et al.*.[1,9] (1*R*,5*R*)-carveol (blue) from our in-house library, synthesis according to Dhulut *et al.*.[1,10] (1*R*,5*S*)-carveol (green) derived from CDO_M232A-catalyzed biotransformation of **1**.[1] (1*R*,5*S*)-carveol (red) derived from CDO wild-type-catalyzed biotransformation of **1**.

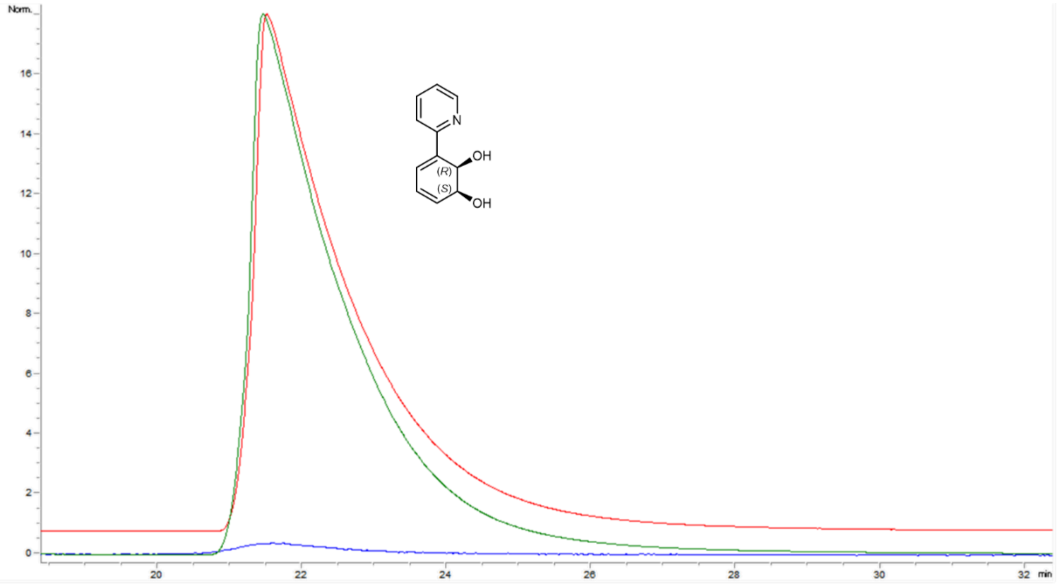

Supplementary Fig. 27: Chiral HPLC analysis of (1*S*,2*R*)-1,2-dihydroxy-3-(2'-pyridyl)-cyclohexa-3,5-diene 3a with normal phase CHIRALPAK IB column. CDO wild-type (blue) and CDO_A283del (red) catalyzed biotransformation of **3**. In-house standard of (1*S*,2*R*)-3a (green).

We also evaluated a normal phase CHIRALPAK IC (250 mm x 4.6 mm, 5 µm particle size, Daicel (Europa) GmbH, Raunheim, Germany) for additional enantiomers of (1S,2R)-1,2-dihydroxy-3-(2'-pyridyl)-cyclohexa-3,5-diene. 6 µl sample were injected and the compounds were separated with 1.4 ml/min isocratic mobile phase of 90:10 n-hexane/isopropanol at 30 °C on the same normal phase HPLC-DAD system mentioned in the methods section. Analysis was performed using the DAD at a wavelength of 310 nm. Despite a length of 90 min and previously successful separation of enantiomers of similar products with this column, no additional peak was observed.

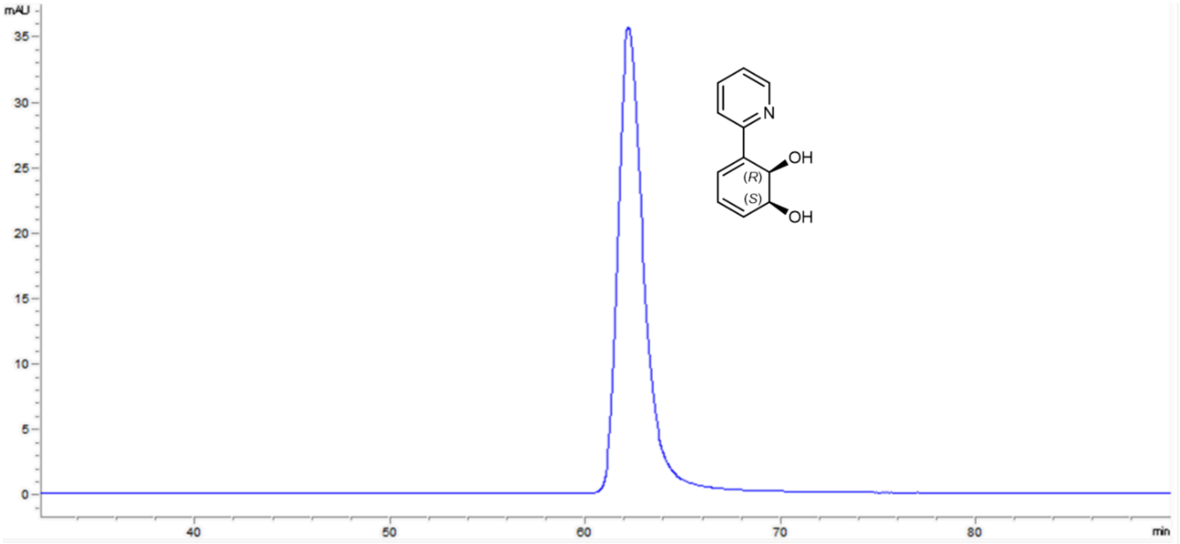

Supplementary Fig. 28: Chiral HPLC analysis of (1*S*,2*R*)-1,2-dihydroxy-3-(2'-pyridyl)-cyclohexa-3,5-diene 3a with normal phase CHIRALPAK IC column. CDO wild-type (blue) and CDO_A283del (red) catalyzed biotransformation of **3**. In-house standard of (1*S*,2*R*)-3a (green).

Primers

Supplementary Table 13: Sequencing primers used in this study.

Name	Sequence (5‘ → 3‘)
**pIP107D_seq1f**	GATATGTACCATGCGGG
**pIP107D_seq2f**	CCAAAATGTACAGCTGTG
**pIP107D_seq3f**	GTGGATTTGCAGGTCGG
**pIP107D_seq4f**	GCCGCTGAAGATATATCCG
**pIP107D_seq5f**	GGTATCGCATGTGAGC

Supplementary Table 14: Site-directed mutagenesis primers used for the alanine scan.

Mutation	Name	Sequence (5‘ → 3‘)
**G236A**	G236A_fw	GATATGTACCATGCGGCAACGATGGCGCATCTT
	G236A_rv	GCGCCATCGTTGCCGCATGGTACATATC
**T237A**	T237A_fw	GCGGGAGCGATGGCGCATCTTTC
	T237A_rv	GAAAGATGCGCCATCGCTCCCGC
**M238A**	M238A_fw	GTACCATGCGGGAACGGCGGCGCATCTTTCAGGT
	M238A_rv	ACCTGAAAGATGCGCCGCCGTTCCCGCATGGTAC
**A239G**	A239G_fw	GGAACGATGGGCCATCTTTCAG
	A239G_rv	CTGAAAGATGGCCCATCGTTCC
**H240A**	H240A_fw	CATGCGGGAACGATGGCGGCTCTTTCAGGTGTATTGTC
	H240A_rv	GACAATACACCTGAAAGAGCCGCCATCGTTCCCGCATG
**L241A**	L241A_fw	GGACAATACACCTGAAGCATGCGCCATCGTTCCCG
	L241A_rv	CGGGAACGATGGCGCATGCTTCAGGTGTATTGTCC
**D242A**	D242A_fw	GGGAACGATGGCGCATCTTGCAGGTGTATTGTCCAGCCTCCCG
	D242A_rv	AAGATGCGCCATCGTTCCCGCATGGTACATATCGCTACAGAATTG
**G243G**	G243G_fw	GAACGATGGCGCATCTTTCAGCTGTATTGTCCAGCCTCCCGCC
	G243G_rv	CTGAAAGATGCGCCATCGTTCCCGCATGGTACATATCGCTACAGAAT
**V244A**	V244A_fw	GATGGCGCATCTTTCAGGTGCATTGTCCAGCCTCCCGCC
	V244A_rv	CACCTGAAAGATGCGCCATCGTTCCCGCATGGTACATATCGC
**L245A**	L245A_fw	GATGGCGCATCTTTCAGGTGTAGCGTCCAGCCTCCCGCCTG
	L245A_rv	TACACCTGAAAGATGCGCCATCGTTCCCGCATGGTACATATCGC
**S246A**	S246A_fw	GCGCATCTTTCAGGTGTATTGGCCAGCCTCCCGCCTGAAATG
	S246A_rv	CAATACACCTGAAAGATGCGCCATCGTTCCCGCATGGTACATATCG
**S247A**	S247A_fw	GCATCTTTCAGGTGTATTGTCCGCCCTCCCGCCTGAAATGGATTTGTC
	S247A_rv	GGACAATACACCTGAAAGATGCGCCATCGTTCCCGCATGGTAC
**L248A**	L248A_fw	GGTGTATTGTCCAGCGCCCCGCCTGAAATGGATT
	L248A_rv	AATCCATTTCAGGCGGGGCGCTGGACAATACACC
**P249A**	P249A_fw	GTATTGTCCAGCCTCGCGCCTGAAATGG
	P249A_rv	CCATTTCAGGCGCGAGGCTGGACAATAC
**P250A**	P250A_fw	TTGTCCAGCCTCCCGGCTGAAATGGATTTGTC
	P250A_rv	GACAAATCCATTTCAGCCGGGAGGCTGG
**E251A**	E251A_fw	CAGCCTCCCGCCTGCAATGGATTTGTCCC
	E251A_rv	GGGACAAATCCATTGCAGGCGGGAGGCTG
**M252A**	M252A_fw	CAGCCTCCCGCCTGAAGCGGATTTGTCCCAAGTAAAG
	M252A_rv	CTTTACTTGGGACAAATCCGCTTCAGGCGGGAGGCTG

**D253A**	D253A_fw	CCTCCCGCCTGAAATGGCTTTGTCCCAAGTAAAG
	D253A_rv	CTTTACTTGGGACAAAGCCATTTCAGGCGGGAGG
**L254A**	L254A_fw	CTCCCGCCTGAAATGGATGCGTCCCAAGTAAAGTTAC
	L254A_rv	GTAACTTTACTTGGGACGCATCCATTTCAGGCGGGAG
**S255A**	S255A_fw	CGCCTGAAATGGATTTGGCCCAAGTAAAGTTACCG
	S255A_rv	GTAACTTTACTTGGGCCAAATCCATTTCAGGCG
**Q256A**	Q256A_fw	CGCCTGAAATGGATTTGTCCGCAGTAAAGTTACCGTCAAGTG
	Q256A_rv	CACTTGACGGTAACTTTACTGCGGACAAATCCATTTCAGGCG
**V257A**	V257A_fw	GGATTTGTCCCAAGCAAAGTTACCGTCAAGTGG
	V257A_rv	CCACTTGACGGTAACTTTGCTTGGGACAAATCCATTTC
**K258A**	K258A_fw	GATTTGTCCCAAGTAGCGTTACCGTCAAGTG
	K258A_rv	CACTTGACGGTAACGCTACTTGGGACAAATC
**L259A**	L259A_fw	CCTGAAATGGATTTGTCCCAAGTAAAGGCACCGTCAAGTGGG
	L259A_rv	CCCACTTGACGGTGCCTTTACTTGGGACAAATCCATTTCAGG
**P260A**	P260A_fw	GATTTGTCCCAAGTAAAGTTAGCGTCAAGTGGG
	P260A_rv	CCCACTTGACGCTAACTTTACTTGGGACAAATC
**S261A**	S261A_fw	CAAGTAAAGTTACCGGCAAGTGGGAATCAG
	S261A_rv	CTGATTCCCACTTGCCGGTAACTTTACTTG
**S262A**	S262A_fw	CCAAGTAAAGTTACCGTCAGCTGGGAATCAGTTCC
	S262A_rv	GAACTGATTCCCAGCTGACGGTAACTTTACTTGGGAC
**G263A**	G263A_fw	GTTACCGTCAAGTGCGAATCAGTTCCGGGC
	G263A_rv	GCCCGGAACTGATTCGCACTTGACGGTAAC
**N264A**	N264A_fw	GTTACCGTCAAGTGGGGCTCAGTTCCGGGCTAAGTG
	N264A_rv	CACTTAGCCCGGAACTGAGCCCCACTTGACGGTAAC
**F278A**	F278A_fw	GACATGGGACCGGCTGGGCCAATGACGATTTCGCAC
	F278A_rv	GTGCGAAATCGTCATTGGCCCAGCCGGTCCCATGTC
**N279A**	N279A_fw	CATGGGACCGGCTGGTTCGCTGACGATTTCGCACTTC
	N279A_rv	GAAGTGCGAAATCGTCAGCGAACCAGCCGGTCCCATG
**D280A**	D280A_fw	CTGGTTCAATGCCGATTTCGCAC
	D280A_rv	GTGCGAAATCGGCATTGAACCAG
**D281A**	D281A_fw	CGGCTGGTTCAATGACGCTTTCGCACTTCTGCAAG
	D281A_rv	CTTGCAGAAGTGCGAAAGCGTCATTGAACCAGCCG
**F282A**	F282A_fw	CAATGACGATGCCGCACTTCTGC
	F282A_rv	GCAGAAGTGCGGCATCGTCATTG
**A283G**	A283G_fw	GTTCAATGACGATTTCGGACTTCTGCAAGCCATC
	A283G_rv	GATGGCTTGCAGAAGTCCGAAATCGTCATTGAAC
**L284A**	L284A_fw	GTTCAATGACGATTTCGCAGCTCTGCAAGCCATCATG
	L284A_rv	GATGGCTTGCAGAGCTGCGAAATCGTCATTGAACCAG
**L285A**	L285A_fw	GACCCATGATGGCTTGCGCAAGTGCGAAATCGTCATTG
	L285A_rv	CAATGACGATTTCGCACTTGCGCAAGCCATCATGGGTC
**Q286A**	Q286A_fw	GACGATTTCGCACTTCTGGCAGCCATCATGGGTCC
	Q286A_rv	GGACCCATGATGGCTGCCAGAAGTGCGAAATCGTC
**A287G**	A287G_fw	CGCACTTCTGCAAGCAATCATGGGTCCTAAGG
	A287G_rv	CCTTAGGACCCATGATTGCTTGCAGAAGTGCG
**I288A**	I288A_fw	CGCACTTCTGCAAGCCGCCATGGGTCCTAAGGTTG
	I288A_rv	CAACCTTAGGACCCATGGCGGCTTGCAGAAGTGCG
**M289A**	M289A_fw	CACTTCTGCAAGCCATCGCGGGTCCTAAGGTTGTCG
	M289A_rv	CGACAACCTTAGGACCCGCGATGGCTTGCAGAAGTG
**G290A**	G290A_fw	CTGCAAGCCATCATGGCTCCTAAGGTTGTCGATTAC
	G290A_rv	CGACAACCTTAGGAGCCATGATGGCTTGCAG

Supplementary Table 15: Site-directed mutagenesis primers used for saturation of selected loop positions

Mutation	Name	Sequence (5‘ → 3‘)
**F278NNK**	F278NNK_fw	CATGGGACCGGCTGGNNKAATGACGATTTCGCAC
	F278NNK_rv	GTGCGAAATCGTCATTMNNCCAGCCGGTCCCATG
**D280NNK**	D280NNK_fw	GACCGGCTGGTTCAATNNKGATTTCGCACTTCTG
	D280NNK_rv	CAGAAGTGCGAAATCMNNATTGAACCAGCCGGTC
**D281NNK**	D281NNK_fw	CGGCTGGTTCAATGACNNKTTCGCACTTCTGCAAG
	D281NNK_rv	CTTGCAGAAGTGCGAAMNNGTCATTGAACCAGCC
**F282NNK**	F282NNK_fw	CTGGTTCAATGACGATNNKGCACTTCTGCAAGCC
	F282NNK_rv	GGCTTGCAGAAGTGCMNNATCGTCATTGAACCAG
**A283NNK**	A283NNK_fw	GTTCAATGACGATTTCNNKCTTCTGCAAGCCATC
	A283NNK_rv	GATGGCTTGCAGAAGMNNGAAATCGTCATTGAAC
**L284NNK**	L284NNK_fw	CAATGACGATTTCGCANNKCTGCAAGCCATCATGG
	L284NNK_rv	CCATGATGGCTTGCAGMNNTGCGAAATCGTCATTG
**L285NNK**	L285NNK_fw	CAATGACGATTTCGCACTTNNKCAAGCCATCATGGGTC
	L285NNK_rv	GACCCATGATGGCTTGMNNAAGTGCGAAATCGTCATTG
**Q286NNK**	Q286NNK_fw	CGATTTCGCACTTCTGNNKGCCATCATGGGTCCTAAG
	Q286NNK_rv	CTTAGGACCCATGATGGCMNNCAGAAGTGCGAAATCG
**A287NNK**	A287NNK_fw	GATTTCGCACTTCTGCAANNKATCATGGGTCCTAAGGTTG
	A287NNK_rv	CAACCTTAGGACCCATGATMNNTTGCAGAAGTGCGAAATC
**I288NNK**	I288NNK_fw	CGCACTTCTGCAAGCCNNKATGGGTCCTAAGGTTGTC
	I288NNK_rv	GACAACCTTAGGACCCATMNNGGCTTGCAGAAGTGCG
**M289NNK**	M289NNK_fw	GCACTTCTGCAAGCCATCNNKGGTCCTAAGGTTGTCG
	M289NNK_rv	CGACAACCTTAGGACCMNNGATGGCTTGCAGAAGTGC

Supplementary Table 16: Site-directed mutagenesis primers used for the adaption library based on the sequence alignment with oxygenases from *Phenylobacterium immobile* E.

Mutation	Name	Sequence (5‘ → 3‘)
**T237del**	T237del_fw	GTACCATGCGGGAACGGCGCATCTTTCAGG
	T237del_rv	CCTGAAAGATGCGCCGTTCCCGCATGGTAC
**M238del**	M238del_fw	GTACCATGCGGGAATGGCGCATCTTTC
	M238del_rv	GAAAGATGCGCCATTCCCGCATGGTAC
**T237I**	T237I_fw	GATATGTACCATGCGGGAATAATGGCGCATC
	T237I_rv	GATGCGCCATTATTCCCGCATGGTACATATC
**A239S**	A239S_fw	CATGCGGGAACGATGTCGCATCTTTCAGGTG
	A239S_rv	CACCTGAAAGATGCGACATCGTTCCCGCATG
**L245M**	L245M_fw	CATCTTTCAGGTGTAATGTCCAGCCTCCCGC
	L245M_rv	GCGGGAGGCTGGACATTACACCTGAAAGATG
**S247G**	S247G_fw	CTTTCAGGTGTATTGTCCGGCCTCCCGCCTGAAATG
	S247G_rv	CATTTCAGGCGGGAGGCCGGACAATACACCTGAAAG
**L248F**	L248F_fw	GGTGTATTGTCCAGCTTCCCGCCTGAAATGGATTTG
	L248F_rv	CAAATCCATTTCAGGCGGGAAGCTGGACAATACACC
**P249R**	P249R_fw	GTGTATTGTCCAGCCTCCGTCCTGAAATGGATTTG
	P249R_rv	CAAATCCATTTCAGGACGGAGGCTGGACAATACAC
**S255L**	S255L_fw	CCTGAAATGGATTTGCTGCAAGTAAAGTTACCG
	S255L_rv	CGGTAACTTTACTTGCAGCAAATCCATTTCAGG
**F266H**	F266H_fw	GTCAAGTGGGAATCAGCACCGGGCTAAGTGGGGTG
	F266H_rv	CACCCCACTTAGCCCGGTGCTGATTCCCACTTGAC

**A268V**	A268V_fw	GAATCAGTTCCGGGTTAAGTGGGGTGGACATG
	A268V_rv	CATGTCCACCCCACTTAACCCGGAACTGATTC
**W270L**	W270L_fw	CAGTTCCGGGCTAAGCTGGGTGGACATGGGACC
	W270L_rv	GGTCCCATGTCCACCCAGCTTAGCCCGGAACTG
**H273Y**	H273Y_fw	GCTAAGTGGGGTGGATATGGGACCGGCTGGTTC
	H273Y_rv	GAACCAGCCGGTCCCATATCCACCCCACTTAGC
**T275H**	T275H_fw	GTGGGGTGGACATGGGCACGGCTGGTTCAATGAC
	T275H_rv	GTCATTGAACCAGCCGTGCCCATGTCCACCCCAC
**G276A**	G276A_fw	GGTGGACATGGGACCGCCTGGTTCAATGACGATTTC
	G276A_rv	GAAATCGTCATTGAACCAGGCGGTCCCATGTCCACC
**W277I**	W277I_fw	GGACATGGGACCGGCATTTTCAATGACGATTTC
	W277I_rv	GAAATCGTCATTGAAAATGCCGGTCCCATGTCC
**F278G**	F278G_fw	CATGGGACCGGCTGGGGCAATGACGATTTCGC
	F278G_rv	GCGAAATCGTCATTGCCCCAGCCGGTCCCATG
**D280E**	D280E_fw	GACCGGCTGGTTCAATGAAGATTTCGCACTTCTG
	D280E_rv	CAGAAGTGCGAAATCTTCATTGAACCAGCCGGTC
**D281E**	D281E_fw	GGCTGGTTCAATGACGAATTCGCACTTCTGCAAG
	D281E_rv	CTTGCAGAAGTGCGAATTCGTCATTGAACCAGCC

Supplementary Table 17: Site-directed insertion mutagenesis primers used for the adaption library based on the sequence alignment with oxygenases from Phenylobacterium immobile E.

Mutation	Name	Sequence (5‘ → 3‘)
**V257_K258 insED**	V257InsED_fw	GGATTTGTCCCAAGTAGAAGACAAGTTACCGTCAAGTGG
	V257InsED_rv	CCACTTGACGGTAACTTGTCTTCTACTTGGGACAAATCC
**V257_K258 insEDQE**	V257InsEDQE_fw	GGATTTGTCCCAAGTAGAAGACCAGGAAAAGTTACCGTCAAGTGG
	V257InsEDQE_rv	CCACTTGACGGTAACTTTTCCTGGTCTTCTACTTGGGACAAATCC
**V257_K258 insEDQELA**	V257InsEDQELA_fw	GGATTTGTCCCAAGTAGAAGACCAGGAACTGGCGAAGTTACCGTCAAGTGG
	V257InsEDQELA_rv	CCACTTGACGGTAACTTCGCCAGTTCCTGGTCTTCTACTTGGGACAAATCC
**V257_K258 insEDQELARI**	V257InsEDQELARI_fw	GGATTTGTCCCAAGTAGAAGACCAGGAACTGGCGCGTATTAAGTTACCGTCAAGTGG
	V257InsEDQELARI_rv	CCACTTGACGGTAACTTAATACGCGCCAGTTCCTGGTCTTCTACTTGGGACAAATCC
**V257_K258 insEDQELARIA**	V257InsEDQELARIA_fw	GGATTTGTCCCAAGTAGAAGACCAGGAACTGGCGCGTATTGCGAAGTTACCGTCAAGTGG
	V257InsEDQELARIA_rv	CCACTTGACGGTAACTTCGCAATACGCGCCAGTTCCTGGTCTTCTACTTGGGACAAATCC
**L259_P260 insG**	L259InsG_fw	GATTTGTCCCAAGTAAAGTTAGGCCCGTCAAGTGGGAATC
	L259InsG_rv	GATTCCCACTTGACGGGCCTAACTTTACTTGGGACAAATC
**P260_S261 insG**	L260InsG_fw	GTCCCAAGTAAAGTTACCGGGCTCAAGTGGGAATCAG
	L260InsG_rv	CTGATTCCCACTTGAGCCCGGTAACTTTACTTGGGAC
**V257_K258 insEE**	V257InsEE_fw	GGATTTGTCCCAAGTAGAAGAAAAGTTACCGTCAAGTGG
	V257InsEE_rv	CCACTTGACGGTAACTTTTCTTCTACTTGGGACAAATCC
**V257_K258 insEERE**	V257InsEERE_fw	GGATTTGTCCCAAGTAGAAGAACGCGAAAAGTTACCGTCAAGTGG
	V257InsEERE_rv	CCACTTGACGGTAACTTTTCGCGTTCTTCTACTTGGGACAAATCC

**V257_K258 insEEREL A**	V257InsEERELA_fw	GGATTTGTCCCAAGTAGAAGAACGCGAACTGGCAAAGTTAC CGTCAAGTGG
	V257InsEERELA_rv	CCACTTGACGGTAACTTTGCCAGTTCGCGTTCTTCTACTTG GGACAAATCC
**V257_K258 insEEREL AA**	V257InsEERELAA_fw	GGATTTGTCCCAAGTAGAAGAACGCGAACTGGCGGCAAAGT TACCGTCAAGTGG
	V257InsEERELAA_rv	CCACTTGACGGTAACTTTGCCGCCAGTTCGCGTTCTTCTAC TTGGGACAAATCC
**K258_L259 insAG**	K258InsAG_fw	GATTTGTCCCAAGTAAAGGCGGGCTTACCGTCAAGTGGG
	K258InsAG_rv	CCCACTTGACGGTAAGCCCGCCTTTACTTGGGACAAATC
**L259_P260 insA**	L259InsA_fw	GATTTGTCCCAAGTAAAGTTAGCGCCGTCAAGTGGGAATC
	L259InsA_rv	GATTCCCACTTGACGGCGCTAACTTTACTTGGGACAAATC
**P260_S261 insGS**	P260InsGS_fw	GTCCCAAGTAAAGTTACCGGGTAGCTCAAGTGGGAATCAG
	P260InsGS_rv	CTGATTCCCACTTGAGCTACCCGGTAACTTTACTTGGGAC
**P260_S261 insGG**	P260InsGG_fw	GTCCCAAGTAAAGTTACCGGGTGGTTCAAGTGGGAATCAG
	P260InsGG_rv	CTGATTCCCACTTGAACCACCCGGTAACTTTACTTGGGAC
**N279_D280 insE**	N279InsE_fw	GGGACCGGCTGGTTCAATAACGACGATTTCGCACTTC
	N279InsE_rv	GAAGTGCGAAATCGTCGTTATTGAACCAGCCGGTCCC
**Q286_A287 insKK**	Q286InsKK_fw	CGATTTCGCACTTCTGCAAAAAAAAAGCCATCATGGGTCCTA AG
	Q286InsKK_rv	CTTAGGACCCATGATGGCTTTTTTTTGCAGAAGTGCGAAAT CG
**Q286_A287 insKKAA**	Q286InsKKAA_fw	CGATTTCGCACTTCTGCAAAAAAAAAGCGGCGGCCATCATGG GTCCTAAG
	Q286InsKKAA_rv	CTTAGGACCCATGATGGCCGCCGCTTTTTTTTGCAGAAGTG CGAAATCG
**Q286_A287 insKKAAE G**	Q286InsKKAAEG_fw	CGATTTCGCACTTCTGCAAAAAAAAAGCGGCGGAAGGCGCCA TCATGGGTCCTAAG
	Q286InsKKAAEG_rv	CTTAGGACCCATGATGGCGCCTTCCGCCGCTTTTTTTTGCA GAAGTGCGAAATCG
**F278_N279 insGP**	F278InsGP_fw	CATGGGACCGGCTGGTTCGGCCCAAATGACGATTTCGCAC
	F278InsGP_rv	GTGCGAAATCGTCATTTGGGCCGAACCAGCCGGTCCCATG
**F278_N279 insGPRL**	F278InsGPRL_fw	CATGGGACCGGCTGGTTCGGCCCACGTCTGAATGACGATTT CGCAC
	F278InsGPRL_rv	GTGCGAAATCGTCATTCAGACGTGGGCCGAACCAGCCGGTC CCATG
**Q286_A287 insEM**	N279InsP_fw	GGGACCGGCTGGTTCAATCCGGACGATTTCGCACTTC
	N279InsP_rv	GAAGTGCGAAATCGTCCGGATTGAACCAGCCGGTCCC
**Q286_A287 insEM**	Q286InsEM_fw	CGATTTCGCACTTCTGCAAGAAATGGCCATCATGGGTCCTA AG
	Q286InsEM_rv	CTTAGGACCCATGATGGCCATTTCTTGCAGAAGTGCGAAAT CG
**Q286_A287 insEMKA**	Q286InsEMKA_fw	CGATTTCGCACTTCTGCAAGAAATGAAAGCCGCCATCATGG GTCCTAAG
	Q286InsEMKA_rv	CTTAGGACCCATGATGGCGGCTTTCATTTCTTGCAGAAGTG CGAAATCG
**Q286_A287 insEMKAE G**	Q286InsEMKAEG_fw	CGATTTCGCACTTCTGCAAGAAATGAAAGCCGAAGGCGCCA TCATGGGTCCTAAG
	Q286InsEMKAEG_rv	CTTAGGACCCATGATGGCGCCTTCGGCTTTCATTTCTTGCA GAAGTGCGAAATCG
**Q286_A287**	Q286InsEMKAEGK_fw	CGATTTCGCACTTCTGCAAGAAATGAAAGCCGAAGGCAAAG CCATCATGGGTCCTAAG

**insEMKAE GK**	Q286InsEMKAEGK_rv	CTTAGGACCCATGATGGCTTTGCCTTCGGCTTTCATTTCTT GCAGAAGTGCGAAATCG

Supplementary Table 18: Site-directed mutagenesis primers used for the deletion variants. * = mutations incorporated by overlap PCR

Mutation	Name	Sequence (5‘ → 3‘)
**E251del**	P250DelE_fw	GTCCAGCCTCCCGCCTATGGATTTGTCCCAAGTAAAGTTACCG
	P250DelE_rv	AGGCGGGAGGCTGGACAATACACCTGAAAGATGCGCCATC
**P250_M252 del**	P249DelPEM_ fw	CAGGTGTATTGTCCAGCCTCCCGGATTTGTCCCAAGTAAAGTTACC
	P249DelPEM_ rv	GGTAACTTTACTTGGGACAAATCCGGGAGGCTGGACAATACACCTG
**L248_L254 del ***	S247DelLPPE MDL_fw	CGCATCTTTCAGGTGTATTGTCCAGCTCCCAAGTAAAGTTACCGTCA AGTGGG
	S247DelLPPE MDL_rv	GCTGGACAATACACCTGAAAGATGCGCCATCGTTCCCGCATGGTACA TATC
**V257del**	Q256DelV_fw	GCCTGAAATGGATTTGTCCCAAAAGTTACCGTCAAGTGGGAATCAGT TC
	Q256DelV_rv	TTGGGACAAATCCATTTCAGGCGGGAGGCTGGACAATACACCTG
**Q256_K258 del**	S255DelQVK_ fw	CGCCTGAAATGGATTTGTCCTTACCGTCAAGTGGGAATCAGTTCCG
	S255DelQVK_ rv	GGACAAATCCATTTCAGGCGGGAGGCTGGACAATACACCTGAAAG
**L254_P260 del ***	D253DelLSQ VKLP_fw	GCCTCCCGCCTGAAATGGATTCAAGTGGGAATCAGTTCCGGGCTAAG
	D253DelLSQ VKLP_rv	ATCCATTTCAGGCGGGAGGCTGGACAATACACCTGAAAGATGCGCC
**A283del**	F282DelA_fw	GGCTGGTTCAATGACGATTTCCTTCTGCAAGCCATCATGGGTC
	F282DelA_rv	GAAATCGTCATTGAACCAGCCGGTCCCATGTCCACCCCAC
**F282_L284 del**	D281DelFAL_ fw	CCGGCTGGTTCAATGACGATCTGCAAGCCATCATGGGTCC
	D281DelFAL_ rv	TCGTCATTGAACCAGCCGGTCCCATGTCCACCCCACTTAGC
**D280_Q286 del ***	N279DelDDF ALLQ_fw	CATGGGACCGGCTGGTTCAATGCCATCATGGGTCCTAAGGTTGTCG
	N279DelDDF ALLQ_rv	ATTGAACCAGCCGGTCCCATGTCCACCCCACTTAGCCCGG

Supplementary Table 19: Site-directed mutagenesis primers used for the LILI library after position V257.

Mutation	Name	Sequence (5' → 3')
**V257_K258 insGG**	V257_K258insGG_fw	GGATTTGTCCCAAGTAGGTGGTAAGTTACCGTCAAGTGG
	V257_K258insGG_rv	CCACTTGACGGTAACTTACCACCTACTTGGGACAAATCC
**V257_K258 insGGG**	V257_K258insGGG_fw	GGATTTGTCCCAAGTAGGTGGTGGTAAGTTACCGTCAAGTGG
	V257_K258insGGG_rv	CCACTTGACGGTAACTTACCACCACCTACTTGGGACAAATCC
**V257_K258 insGGGG**	V257_K258insGGGG_fw	GGATTTGTCCCAAGTAGGTGGTGGTGGTAAGTTACCGTCAAGTGG
	V257_K258insGGGG_rv	CCACTTGACGGTAACTTACCACCACCACCTACTTGGGACAAATCC
**V257_K258 insGGGGG**	V257_K258insGGGGG_fw	GGATTTGTCCCAAGTAGGTGGTGGTGGTGGTAAGTTACCGTCAAGTGG
	V257_K258insGGGGG_rv	CCACTTGACGGTAACTTACCACCACCACCACCTACTTGGGACAAATCC
**V257_K258 insGGGGGG**	V257_K258insGGGGGG_fw	GGATTTGTCCCAAGTAGGTGGTGGTGGTGGTGGTAAGTTACCGTCAAGTGG
	V257_K258insGGGGGG_rv	CCACTTGACGGTAACTTACCACCACCACCACCACCTACTTGGGACAAATCC
**V257_K258 insGS**	V257_K258insGS_fw	GGATTTGTCCCAAGTAGGTTCTAAGTTACCGTCAAGTGG
	V257_K258insGS_rv	CCACTTGACGGTAACTTAGAACCTACTTGGGACAAATCC
**V257_K258 insGSG**	V257_K258insGSG_fw	GGATTTGTCCCAAGTAGGTTCTGGTAAGTTACCGTCAAGTGG
	V257_K258insGSG_rv	CCACTTGACGGTAACTTAGAACCAGATACTTGGGACAAATCC
**V257_K258 insGSGS**	V257_K258insGSGS_fw	GGATTTGTCCCAAGTAGGTTCTGGTTCTAAGTTACCGTCAAGTGG
	V257_K258insGSGS_rv	CCACTTGACGGTAACTTAGAACCAGAACCTACTTGGGACAAATCC
**V257_K258 insGSGSG**	V257_K258insGSGSG_fw	GGATTTGTCCCAAGTAGGTTCTGGTTCTGGTAAGTTACCGTCAAGTGG
	V257_K258insGSGSG_rv	CCACTTGACGGTAACTTAGAACCAGAACCAGATACTTGGGACAAATCC
**V257_K258 insGSGSGS**	V257_K258insGSGSGS_fw	GGATTTGTCCCAAGTAGGTTCTGGTTCTGGTTCTAAGTTACCGTCAAGTGG
	V257_K258insGSGSGS_rv	CCACTTGACGGTAACTTAGAACCAGAACCAGAACCTACTTGGGACAAATCC
**V257_K258 insPA**	V257_K258insPA_fw	GGATTTGTCCCAAGTACCGGCAAAGTTACCGTCAAGTGG
	V257_K258insPA_rv	CCACTTGACGGTAACTTTGCCGGTACTTGGGACAAATCC
**V257_K258 insPAP**	V257_K258insPAP_fw	GGATTTGTCCCAAGTACCGGCACCGAAGTTACCGTCAAGTGG
	V257_K258insPAP_rv	CCACTTGACGGTAACTTCGGTGCCGGTACTTGGGACAAATCC

**V257_K258 insPAPA**	V257_K258insPAPA_fw	GGATTTGTCCCAAGTACCGGCACCGGCAAAGTTACCGTCAAGTGG
	V257_K258insPAPA_rv	CCACTTGACGGTAACTTTGCCGGTGCCGGTACTTGGGACAAATCC
**V257_K258 insPAPAP**	V257_K258insPAPAP_fw	GGATTTGTCCCAAGTACCGGCACCGGCACCGAAGTTACCGTCAAGTGG
	V257_K258insPAPAP_rv	CCACTTGACGGTAACTTCGGTGCCGGTGCCGGTACTTGGGACAAATCC
**V257_K258 insPAPAPA**	V257_K258insPAPAPA_fw	GGATTTGTCCCAAGTACCGGCACCGGCACCGGCAAAGTTACCGTCAAGTGG
	V257_K258insPAPAPA_rv	CCACTTGACGGTAACTTTGCCGGTGCCGGTGCCGGTACTTGGGACAAATCC
**V257_K258 insGP**	V257_K258insGP_fw	GGATTTGTCCCAAGTAGGTCCGAAGTTACCGTCAAGTGG
	V257_K258insGP_rv	CCACTTGACGGTAACTTCGGACCTACTTGGGACAAATCC
**V257_K258 insGPG**	V257_K258insGPG_fw	GGATTTGTCCCAAGTAGGTCCGGGTAAGTTACCGTCAAGTGG
	V257_K258insGPG_rv	CCACTTGACGGTAACTTACCCGGACCTACTTGGGACAAATCC
**V257_K258 insGPGP**	V257_K258insGPGP_fw	GGATTTGTCCCAAGTAGGTCCGGGTCCGAAGTTACCGTCAAGTGG
	V257_K258insGPGP_rv	CCACTTGACGGTAACTTCGGACCCGGACCTACTTGGGACAAATCC
**V257_K258 insGPGPG**	V257_K258insGPGPG_fw	GGATTTGTCCCAAGTAGGTCCGGGTCCGGGTAAGTTACCGTCAAGTGG
	V257_K258insGPGPG_rv	CCACTTGACGGTAACTTACCCGGACCCGGACCTACTTGGGACAAATCC
**V257_K258 insGPGPGP**	V257_K258insGPGPGP_fw	GGATTTGTCCCAAGTAGGTCCGGGTCCGGGTCCGAAGTTACCGTCAAGTGG
	V257_K258insGPGPGP_rv	CCACTTGACGGTAACTTCGGACCCGGACCCGGACCTACTTGGGACAAATCC

Supplementary Table 20: Site-directed mutagenesis primers used for the LILI library after position F278.

Mutation	Name	Sequence (5‘ → 3‘)
**F278_N279 insGG**	F278_N279insGG_fw	GGACATGGGACCGGCTGGTTCGGTGGTAATGACGATTTCGCACTTC
	F278_N279insGG_rv	GAAGTGCGAAATCGTCATTACCACCGAACCAGCCGGTCCCATGTCC
**F278_N279 insGGG**	F278_N279insGGG_fw	GGACATGGGACCGGCTGGTTCGGTGGTGGTAATGACGATTTCGCACTTC
	F278_N279insGGG_rv	GAAGTGCGAAATCGTCATTACCACCACCGAACCAGCCGGTCCCATGTCC
**F278_N279 insGGGG**	F278_N279insGGGG_fw	GGACATGGGACCGGCTGGTTCGGTGGTGGTGGTAATGACGATTTCGCACTTC
	F278_N279insGGGG_rv	GAAGTGCGAAATCGTCATTACCACCACCACCGAACCAGCCGGTCCCATGTCC
**F278_N279 insGGGGG**	F278_N279insGGGGG_fw	GGACATGGGACCGGCTGGTTCGGTGGTGGTGGTGGTAATGACGATTTCGCACTTC
	F278_N279insGGGGG_rv	GAAGTGCGAAATCGTCATTACCACCACCACCACCGAACCAGCCGGTCCCATGTCC
**F278_N279 insGGGGGG**	F278_N279insGGGGGG_fw	GGACATGGGACCGGCTGGTTCGGTGGTGGTGGTGGTGGTAATGACGATTTCGCACTTC
	F278_N279insGGGGGG_rv	GAAGTGCGAAATCGTCATTACCACCACCACCACCACCGAACCAGCCGGTCCCATGTCC
**F278_N279 insGS**	F278_N279insGS_fw	GGACATGGGACCGGCTGGTTCGGTTCTAATGACGATTTCGCACTTC
	F278_N279insGS_rv	GAAGTGCGAAATCGTCATTAGAACCGAACCAGCCGGTCCCATGTCC
**F278_N279 insGSG**	F278_N279insGSG_fw	GGACATGGGACCGGCTGGTTCGGTTCTGGTAATGACGATTTCGCACTTC
	F278_N279insGSG_rv	GAAGTGCGAAATCGTCATTACCAGAACCGAACCAGCCGGTCCCATGTCC
**F278_N279 insGSGS**	F278_N279insGSGS_fw	GGACATGGGACCGGCTGGTTCGGTTCTGGTTCTAATGACGATTTCGCACTTC
	F278_N279insGSGS_rv	GAAGTGCGAAATCGTCATTAGAACCAGAACCGAACCAGCCGGTCCCATGTCC
**F278_N279 insGSGSG**	F278_N279insGSGSG_fw	GGACATGGGACCGGCTGGTTCGGTTCTGGTTCTGGTAATGACGATTTCGCACTTC
	F278_N279insGSGSG_rv	GAAGTGCGAAATCGTCATTACCAGAACCAGAACCGAACCAGCCGGTCCCATGTCC
**F278_N279 insGSGSGS**	F278_N279insGSGSGS_fw	GGACATGGGACCGGCTGGTTCGGTTCTGGTTCTGGTTCTAATGACGATTTCGCACTTC
	F278_N279insGSGSGS_rv	GAAGTGCGAAATCGTCATTAGAACCAGAACCAGAACCGAACCAGCCGGTCCCATGTCC
**F278_N279 insPA**	F278_N279insPA_fw	GGACATGGGACCGGCTGGTTCCCGGCAAATGACGATTTCGCACTTC
	F278_N279insPA_rv	GAAGTGCGAAATCGTCATTTGCCGGGAACCAGCCGGTCCCATGTCC
**F278_N279 insPAP**	F278_N279insPAP_fw	GGACATGGGACCGGCTGGTTCCCGGCACCGAATGACGATTTCGCACTTC
	F278_N279insPAP_rv	GAAGTGCGAAATCGTCATTCGGTGCCGGGAACCAGCCGGTCCCATGTCC

**F278_N279 insPAPA**	F278_N279insPAPA_fw	GGACATGGGACCGGCTGGTTCCCGGCACCGGCAAATGACGATTTCGCACTTC
	F278_N279insPAPA_rv	GAAGTGCGAAATCGTCATTTGCCGGTGCCGGGAACCAGCCGGTCCCATGTCC
**F278_N279 insPAPAP**	F278_N279insPAPAP_fw	GGACATGGGACCGGCTGGTTCCCGGCACCGGCACCGAATGACGATTTCGCACTTC
	F278_N279insPAPAP_rv	GAAGTGCGAAATCGTCATTCGGTGCCGGTGCCGGGAACCAGCCGGTCCCATGTCC
**F278_N279 insPAPAPA**	F278_N279insPAPAPA_fw	GGACATGGGACCGGCTGGTTCCCGGCACCGGCACCGGCAAATGACGATTTCGCACTTC
	F278_N279insPAPAPA_rv	GAAGTGCGAAATCGTCATTTGCCGGTGCCGGTGCCGGGAACCAGCCGGTCCCATGTCC
**F278_N279 insGP**	F278_N279insGP_fw	GGACATGGGACCGGCTGGTTCGGTCCGAATGACGATTTCGCACTTC
	F278_N279insGP_rv	GAAGTGCGAAATCGTCATTCGGACCGAACCAGCCGGTCCCATGTCC
**F278_N279 insGPG**	F278_N279insGPG_fw	GGACATGGGACCGGCTGGTTCGGTCCGGGTAATGACGATTTCGCACTTC
	F278_N279insGPG_rv	GAAGTGCGAAATCGTCATTACCCGGACCGAACCAGCCGGTCCCATGTCC
**F278_N279 insGPGP**	F278_N279insGPGP_fw	GGACATGGGACCGGCTGGTTCGGTCCGGGTCCGAATGACGATTTCGCACTTC
	F278_N279insGPGP_rv	GAAGTGCGAAATCGTCATTCGGACCCGGACCGAACCAGCCGGTCCCATGTCC
**F278_N279 insGPGPG**	F278_N279insGPGPG_fw	GGACATGGGACCGGCTGGTTCGGTCCGGGTCCGGGTAATGACGATTTCGCACTTC
	F278_N279insGPGPG_rv	GAAGTGCGAAATCGTCATTACCCGGACCCGGACCGAACCAGCCGGTCCCATGTCC
**F278_N279 insGPGPGP**	F278_N279insGPGPGP_fw	GGACATGGGACCGGCTGGTTCGGTCCGGGTCCGGGTCCGAATGACGATTTCGCACTTC
	F278_N279insGPGPGP_rv	GAAGTGCGAAATCGTCATTCGGACCCGGACCCGGACCGAACCAGCCGGTCCCATGTCC

Supplementary Table 21: Site-directed mutagenesis primers used for the LILI library after position Q286.

Mutation	Name	Sequence (5' → 3')
**Q286_A287 insGG**	Q286_A287insGG_fw	GACGATTTCGCACTTCTGCAAGGTGGTGCCATCATGGGTCCTAAGG
	Q286_A287insGG_rv	CCTTAGGACCCATGATGGCACCACCTTGCAGAAGTGCGAAATCGTC
**Q286_A287 insGGG**	Q286_A287insGGG_fw	GACGATTTCGCACTTCTGCAAGGTGGTGGTGCCATCATGGGTCCTAAGG
	Q286_A287insGGG_rv	CCTTAGGACCCATGATGGCACCACCACCTTGCAGAAGTGCGAAATCGTC
**Q286_A287 insGGGG**	Q286_A287insGGGG_fw	GACGATTTCGCACTTCTGCAAGGTGGTGGTGGTGCCATCATGGGTCCTAAGG
	Q286_A287insGGGG_rv	CCTTAGGACCCATGATGGCACCACCACCACCTTGCAGAAGTGCGAAATCGTC
**Q286_A287 insGGGGG**	Q286_A287insGGGGG_fw	GACGATTTCGCACTTCTGCAAGGTGGTGGTGGTGGTGCCATCATGGGTCCTAAGG
	Q286_A287insGGGGG_rv	CCTTAGGACCCATGATGGCACCACCACCACCACCTTGCAGAAGTGCGAAATCGTC
**Q286_A287 insGGGGGG**	Q286_A287insGGGGGG_fw	GACGATTTCGCACTTCTGCAAGGTGGTGGTGGTGGTGGTGCCATCATGGGTCCTAAGG
	Q286_A287insGGGGGG_rv	CCTTAGGACCCATGATGGCACCACCACCACCACCACCTTGCAGAAGTGCGAAATCGTC
**Q286_A287 insGS**	Q286_A287insGS_fw	GACGATTTCGCACTTCTGCAAGGTTCTGCCATCATGGGTCCTAAGG
	Q286_A287insGS_rv	CCTTAGGACCCATGATGGCAGAACCTTGCAGAAGTGCGAAATCGTC
**Q286_A287 insGSG**	Q286_A287insGSG_fw	GACGATTTCGCACTTCTGCAAGGTTCTGGTGCCATCATGGGTCCTAAGG
	Q286_A287insGSG_rv	CCTTAGGACCCATGATGGCACCAGAACCTTGCAGAAGTGCGAAATCGTC
**Q286_A287 insGSGS**	Q286_A287insGSGS_fw	GACGATTTCGCACTTCTGCAAGGTTCTGGTTCTGCCATCATGGGTCCTAAGG
	Q286_A287insGSGS_rv	CCTTAGGACCCATGATGGCAGAACCAGAACCTTGCAGAAGTGCGAAATCGTC
**Q286_A287 insGSGSG**	Q286_A287insGSGSG_fw	GACGATTTCGCACTTCTGCAAGGTTCTGGTTCTGGTGCCATCATGGGTCCTAAGG
	Q286_A287insGSGSG_rv	CCTTAGGACCCATGATGGCACCAGAACCAGAACCTTGCAGAAGTGCGAAATCGTC
**Q286_A287 insGSGSGS**	Q286_A287insGSGSGS_fw	GACGATTTCGCACTTCTGCAAGGTTCTGGTTCTGGTTCTGCCATCATGGGTCCTAAGG
	Q286_A287insGSGSGS_rv	CCTTAGGACCCATGATGGCAGAACCAGAACCAGAACCTTGCAGAAGTGCGAAATCGTC
**Q286_A287 insPA**	Q286_A287insPA_fw	GACGATTTCGCACTTCTGCAACCGGCAGCCATCATGGGTCCTAAGG
	Q286_A287insPA_rv	CCTTAGGACCCATGATGGCTGCCGGTTGCAGAAGTGCGAAATCGTC
**Q286_A287 insPAP**	Q286_A287insPAP_fw	GACGATTTCGCACTTCTGCAACCGGCACCGGCCATCATGGGTCCTAAGG
	Q286_A287insPAP_rv	CCTTAGGACCCATGATGGCCGGTGCCGGTTGCAGAAGTGCGAAATCGTC

**Q286_A287 insPAPA**	Q286_A287insPAPA_fw	GACGATTTCGCACTTCTGCAACCGGCACCGGCAGCCATCATGGGTCCTAAGG
	Q286_A287insPAPA_rv	CCTTAGGACCCATGATGGCTGCCGGTGCCGGTTGCAGAAGTGCGAAATCGTC
**Q286_A287 insPAPAP**	Q286_A287insPAPAP_fw	GACGATTTCGCACTTCTGCAACCGGCACCGGCACCGGCCATCATGGGTCCTAAGG
	Q286_A287insPAPAP_rv	CCTTAGGACCCATGATGGCCGGTGCCGGTGCCGGTTGCAGAAGTGCGAAATCGTC
**Q286_A287 insPAPAPA**	Q286_A287insPAPAPA_fw	GACGATTTCGCACTTCTGCAACCGGCACCGGCACCGGCAGCCATCATGGGTCCTAAGG
	Q286_A287insPAPAPA_rv	CCTTAGGACCCATGATGGCTGCCGGTGCCGGTGCCGGTTGCAGAAGTGCGAAATCGTC
**Q286_A287 insGP**	Q286_A287insGP_fw	GACGATTTCGCACTTCTGCAAGGTCCGGCCATCATGGGTCCTAAGG
	Q286_A287insGP_rv	CCTTAGGACCCATGATGGCCGGACCTTGCAGAAGTGCGAAATCGTC
**Q286_A287 insGPG**	Q286_A287insGPG_fw	GACGATTTCGCACTTCTGCAAGGTCCGGGTGCCATCATGGGTCCTAAGG
	Q286_A287insGPG_rv	CCTTAGGACCCATGATGGCACCCGGACCTTGCAGAAGTGCGAAATCGTC
**Q286_A287 insGPGP**	Q286_A287insGPGP_fw	GACGATTTCGCACTTCTGCAAGGTCCGGGTCCGGCCATCATGGGTCCTAAGG
	Q286_A287insGPGP_rv	CCTTAGGACCCATGATGGCCGGACCCGGACCTTGCAGAAGTGCGAAATCGTC
**Q286_A287 insGPGPG**	Q286_A287insGPGPG_fw	GACGATTTCGCACTTCTGCAAGGTCCGGGTCCGGGTGCCATCATGGGTCCTAAGG
	Q286_A287insGPGPG_rv	CCTTAGGACCCATGATGGCACCCGGACCCGGACCTTGCAGAAGTGCGAAATCGTC
**Q286_A287 insGPGPGP**	Q286_A287insGPGPGP_fw	GACGATTTCGCACTTCTGCAAGGTCCGGGTCCGGGTCCGGCCATCATGGGTCCTAAGG
	Q286_A287insGPGPGP_rv	CCTTAGGACCCATGATGGCCGGACCCGGACCCGGACCTTGCAGAAGTGCGAAATCGTC

NMR spectra

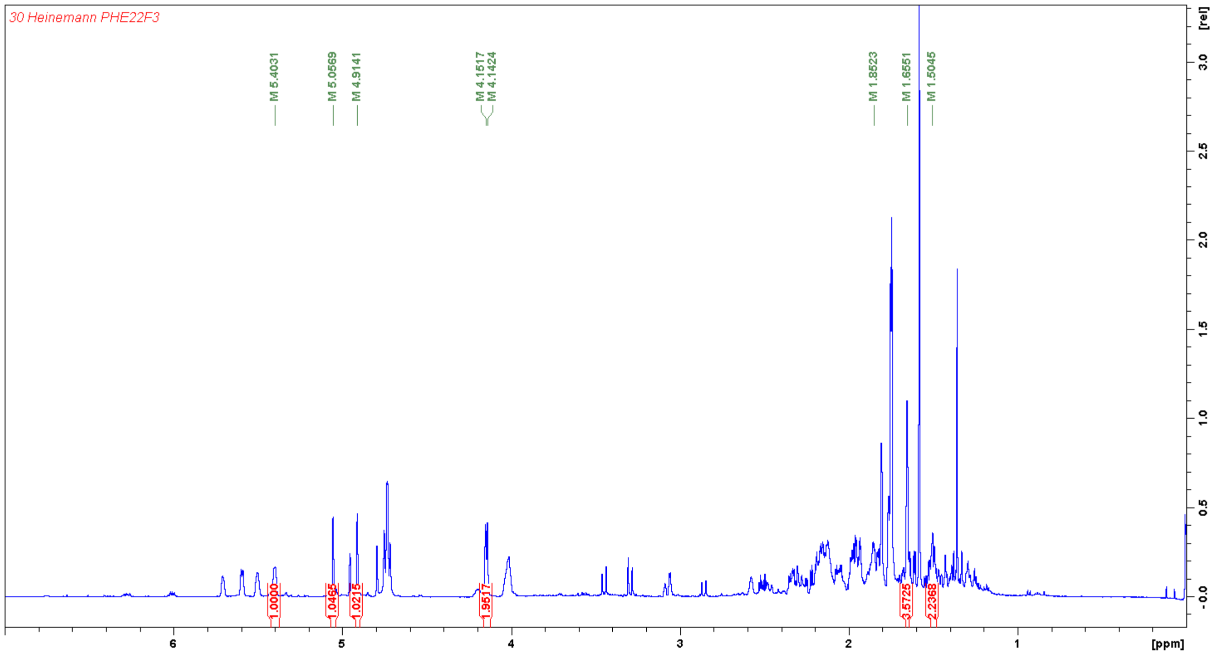

Supplementary Fig. 29: ^{1}H-NMR of the product of the synthesis of (+)-mentha-1.8-dien-10-ol 2b in $CDCl_3$

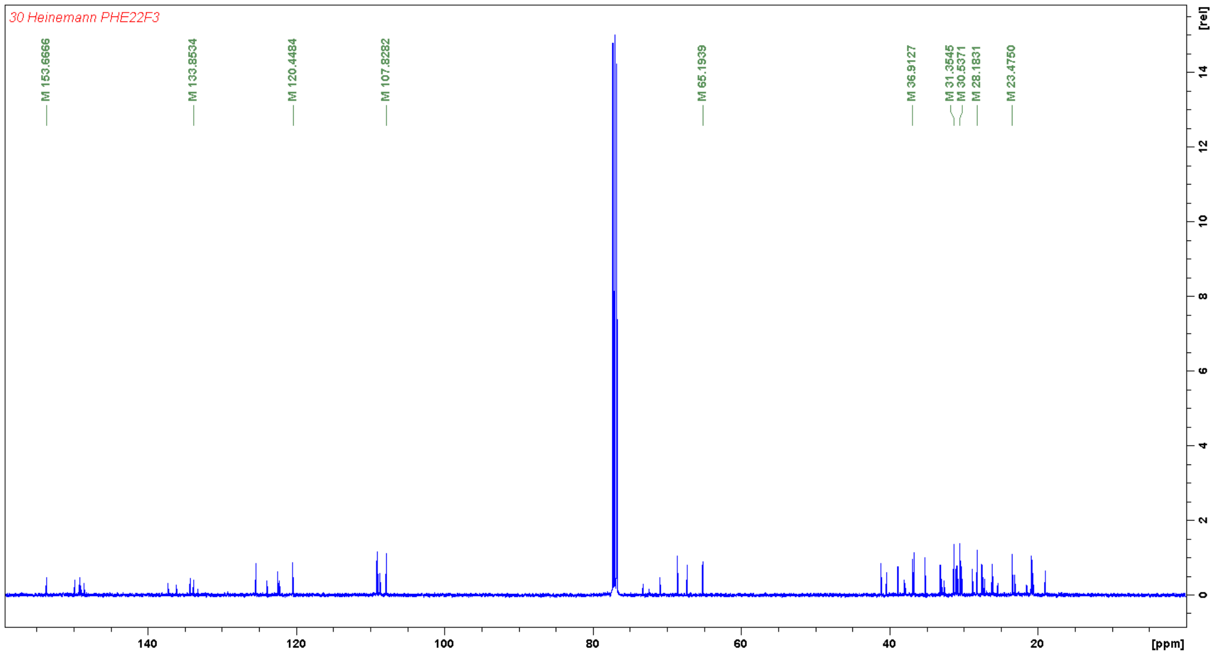

Supplementary Fig. 30: ^{13}C-NMR of the product of the synthesis of (+)-mentha-1.8-dien-10-ol 2b in $CDCl_3$

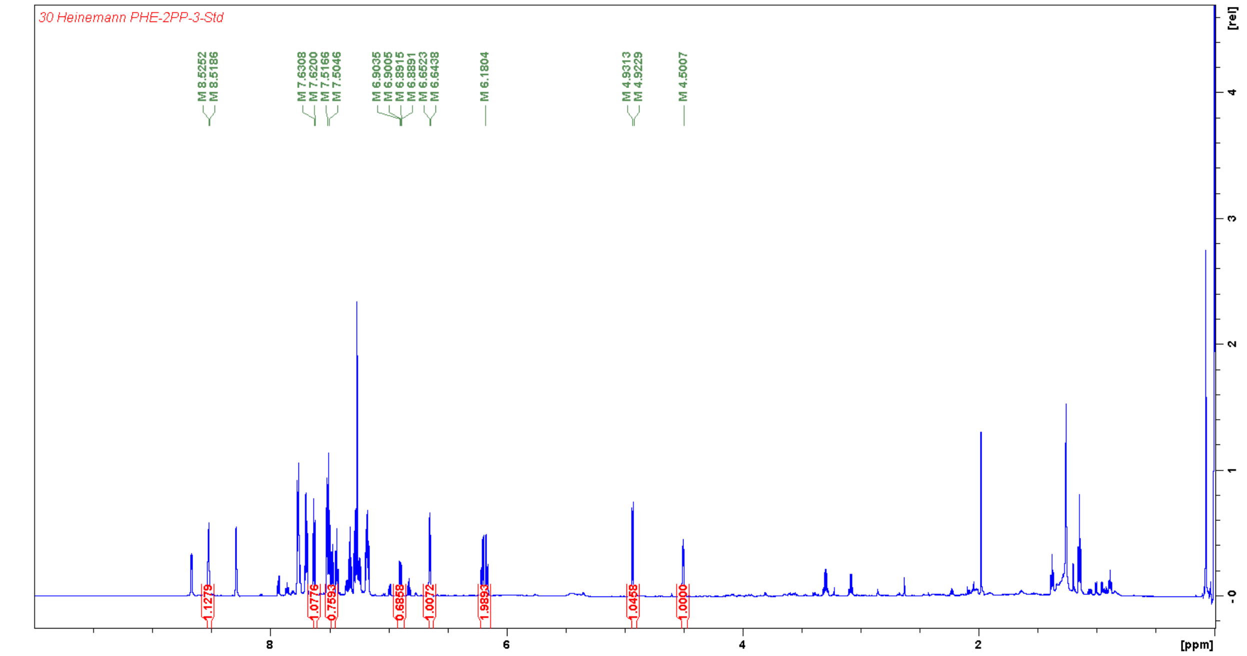

Supplementary Fig. 31: ^{1}H-NMR of the in-house standard of 1,2-dihydroxy-3-(2’-pyridyl)-cyclohexa-3,5-diene 3a.

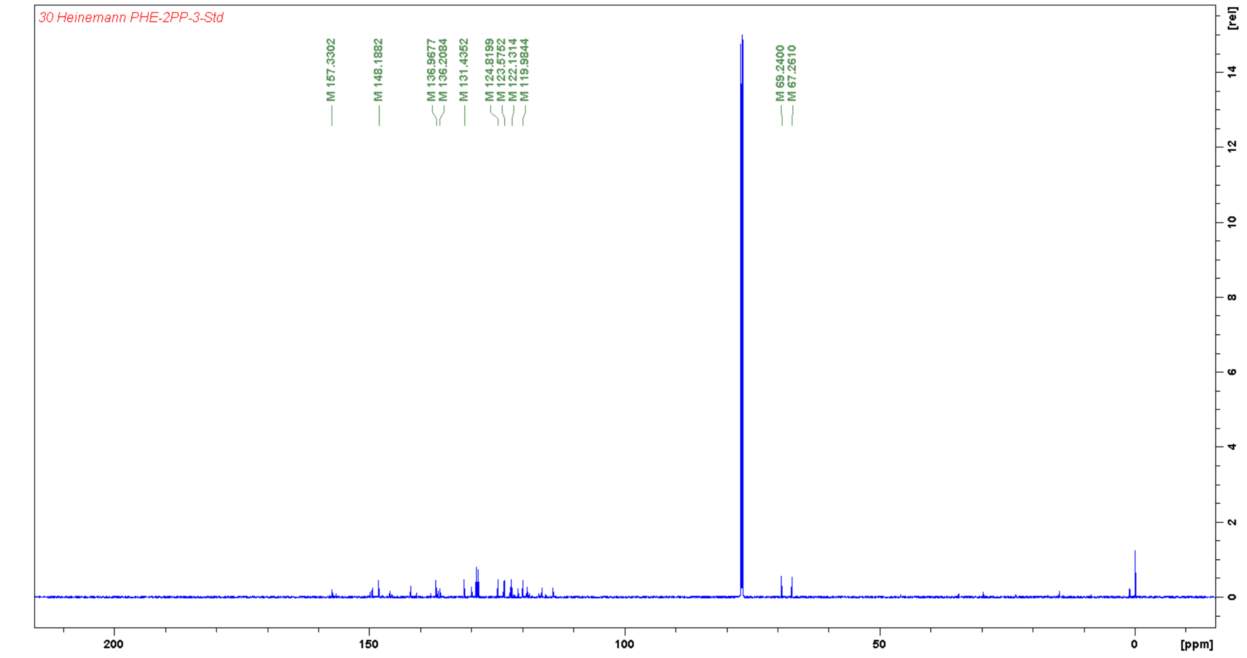

Supplementary Fig. 32: ^{13}C-NMR of the in-house standard of 1,2-dihydroxy-3-(2’-pyridyl)-cyclohexa-3,5-diene 3a.

Sequence of the Cumene Dioxygenase from *Pseudomonas fluorescens* IP01

CDO gene cluster (GenBank: D37828.1)[11]

cumA1 (Oxygenase α-subunit) → cumA2 (Oxygenase β-subunit)→ cumA3 (Ferredoxin) → cumA4 (Reductase)

Overlapping sequence space between cumA3 and cumA4 is underlined.

ATGAGTTCAATAATAAATAAAGAAGTGCAGGAAGCCCCTTTGAAATGGGTGAAAAACTGGTCTGACGAGGAGATTAAAGCGCT
CGTTGATGAGGAAAAGGGGTTGCTTGATCCACGTATTTTCTCTGATCAGGATTTGTATGAGATCGAGCTTGAGAGGGTGTTTG
CTCGATCCTGGCTGCTGCTTGGGCACGAGGGGCACATTCCCAAAGCCGGGGATTATCTGACCACCTACATGGGTGAAGACCCA
GTAATTGTAGTGAGGCAGAAAGACCGGAGCATTAAAGTCTTTTTAAACCAATGTCGGCATCGCGGTATGCGTATTGAGCGATC
GGATTTTGGCAACGCAAAGTCATTTACCTGCACTTATCACGGGTGGGCCTATGACACCGCCGGTAATCTGGTCAATGTACCCT
ACGAGAAAGAGGCTTTTTGTGACAAAAAAGAGGGTGACTGCGGGTTCGACAAGGCCGACTGGGGGCCGCTGCAAGCGCGGGTG
GATACTTACAAGGGGCTGATTTTTGCCAACTGGGATACCGAAGCCCCTGATTTGAAGACCTATCTGAGCGATGCAACACCCTA
TATGGACGTGATGCTCGATCGGACCGAGGCAGTTACTCAGGTCATCACCGGTATGCAAAAGACGGTAATCCCCTGTAACTGGA
AATTCGCCGCCGAGCAATTCTGTAGCGATATGTACCATGCGGGAACGATGGCGCATCTTTCAGGTGTATTGTCCAGCCTCCCG
CCTGAAATGGATTTGTCCCAAGTAAAGTTACCGTCAAGTGGGAATCAGTTCCGGGCTAAGTGGGGTGGACATGGGACCGGCTG
GTTCAATGACGATTTCGCACTTCTGCAAGCCATCATGGGTCCTAAGGTTGTCGATTACTGGACCAAAGGTCCAGCTGCTGAGC
GTGCAAAAGAGCGTCTGGGTAAAGTTCTTCCGGCTGATCGCATGGTTGCTCAGCATATGACCATTTTTCCGACATGCTCATTT
CTTCCTGGCATCAATACAGTCCGTACTTGGCACCCACGTGGCCCTAATGAGATCGAAGTTTGGTCTTTCATCGTAGTGGATGC
TGATGCACCTGAAGATATCAAGGAAGAATATCGTCGGAAAAACATCTTCACCTTCAATCAAGGGGGAACCTACGAGCAGGACG
ATGGCGAAAACTGGGTGGAGGTTCAGCGGGGATTGCGCGGCTACAAGGCTAGAAGTAGACCTCTTTGTGCCCAGATGGGGGCG
GGTGTGCCAAACAAGAACAACCCGGAGTTTCCTGGAAAGACCAGCTACGTTTATAGCGAAGAAGCTGCGCGAGGGTTCTACCA
CCACTGGAGCCGCATGATGTCCGAGCCGAGTTGGGACACGCTAAAGTCTTGAGCAGATAAAGTGACCGAAAAAAGCAATCACT
TTCATCGGGTTTCTACCGTGGTAGACAAGGGTTTAGCCTGTTTTTTGGTTGCTGGAAGTGCCTAAGTGAATTGATTAACTTGG
GTAAACCCCTGGCTTTGTCGGGGGTATTTACTCGGGTGCATTCCAAAATGTACAGCTGTGCGTTTGGTGATAATCGTCATGCT
ATGGATTTGCTATTTGCATGAGCCGAGTGCAGGTCGCCCAACATATATACAGGAAACTAATTATGACATCCGCTGATTTGACA
AAACCCATCGAGTGGCCAGAAATGCCCGTCAGTCTTGAATTGCAAAATGCCGTTGAGCAATTCTACTATCGCGAAGCACAGTT
GCTTGATTATCAAAACTATGAGGCCTGGCTGGCTTTACTGACCCAAGACATCCAATATTGGATGCCAATTCGTACTACTCATA
CATCCCGGAATAAGGCGATGGAGTACGTGCCCCCCGGCGGTAATGCCCATTTTGACGAGACGTATGAGAGCATGCGTGCGCGC
ATTCGGGCGAGGGTTTCGGGGCTTAACTGGACTGAAGATCCACCGTCGCGCAGCCGGCACATTGTAAGCAACGTTATCGTCCG
CGAAACTGAGAGTGCTGGTACTTTGGAAGTTAGTTCTGCGTTCCTTTGTTACCGTAATCGATTGGAGCGTATGACGGACATCT
ATGTCGGTGAGCGTCGAGATATTTTGCTCCGTGTAAGTGACGGGCTGGGATTCAAAATTGCCAAGCGAACGATCTTGCTCGAC
CAGAGCACGATTACAGCGAATAATCTCAGCCAGTTTTTCTAACTAGGGAATGCTGGCCACTTACCCTATACCCAGCCTATTCA
TGAGAGCGGCCTGAAAATGAAGAGGAGCTACCCGATAGCTACGCAAACTAATCGCGCTCGCCCTTTCCTGATCGCGATCGGTA
TCTTTTACTTGGCCAATCTCCTCGGGACTTTGCATTTCAGCAGCCTGCGGCTGTTCGGCATGATGTATTCGGGTGTGGATTTG
CAGGTCGGCGCTCCGGTATTCACCCTGCTGCAGGATGCCTGGGCCGTAGTCGGGCTGCAGCTGGGGGGCACTGGGCTGGTTGC
GTTGTGGGGCGCACGTCAGCCCGTGCGCTTCATGGCGGTTGTCCCCGTGGTCATCGTCACGGAAGTGCTCGACGGTATCTGGG
ACTTGTACAGCATCGTTTGGAGTCACGAAGCCATGTGGTTCGGGCTCCTGACGTTCGCCATCCACGTGGTGTGGATCGTCTGG
GGGTTACAGGTATGGCGCGTGTCGTCTCGCCGGTCATCTGGCTTAACCGTCCCAACCTCCTGAATCTGTGGGCCTGAATTGAA
CTCTGTCATTTCCCATGCGCGTCGACGTTATCCGGCCGCTTGCGCCAAGCTGGCTGCCGGATTTAATGAGTAGTTAAAGTTAG
CTATAGAAACTCTGAAAAAGGCTTGACCTCATGAGATATCCAGTCTGCAGTCCGCGTGGTTACTGGCGTGCATTTTCCGAGTG
CGTACTTTTTCAGACCAACTCTATAATAAGAGACAAAAAAGAATGACTTTTTCCAAAGTTTGTGAAGTATCTGATGTGCCCGT
CGGTGACGCCTTGCAGGTTGAAAGTAAGGGCGAAGCCGTCGCGATTTTCAACGTCGATGGAGAGTTGTTCGCAACACAGGACC
GTTGCACTCATGGTGACTGGTCCTTGTCCGAAGGCGGCTACCTAGAGGGTGACATTGTCGAATGCTCGCTGCACATGGGTAGG
TTCTGTGTCCGCACGGGCAAGGTAAAAGCAGCACCGCCCTGTGAGCCGCTGAAGATATATCCGATTCGAATAGATGGCAGCGA
TGTGTTCGTAGACTTTGATGCCGGGTATCTAGCGCCATGATTAAATCAATCGTCATTATTGGTGCTGGCTTGGCTGGCGCAAC
TGCCACTCGCTATCTTCGCGCCCAAGGATATCAGGGAAAGATCCATCTGGTCGGGGAGGAGTTGCATGTGGCTTACGATCGCC
CCTCCTTATCCAAGGACACCCTGTCAGGAAAAGTGGTCGAACCACCCGCAATCCTGGATCCTTGTTGGTATGCATCGGCCGAT
ATAGATCTCCATTTAGGTGTACGCGTGACCGGTATTGATGTGGTAAACCACCAGGTACTTTTCGAATCCGGTGACATTCTAGC
CTACGACCGACTGCTATTAGCCACCGGCGCTCGCGCGCGGCGTATGGCTATTACGGGAAGCGAGTTGGCCGGCATTCACACCT
TGCGTGACCGCGCCGACAGCCAGGCGCTGAGGCAGGCGCTTGAGCCGGGCCAGTCTCTGGTAATTGTCGGCGGTGGCCTGATC
GGTTGCGAAGTGGCGACCACTGCTATTAATGCCGGTGCCCACGTCACTGTTCTGGAGGCCGGGGACGAACTGCTGTTGCGAGT
GCTAGGCCGATCAACCGGGGCCTGGTGTCGCAACGAGTTGGAGCGTTTGGGTGTCCGGGTTGAACTGAACGCACAGGCAGCGC
ATTTCGAGGGCGAGGGACACGTGCATGCCGTCGTTTGTGCCGATGGACGTCGGATAGCAGCTGGCACAGTTTTGGTGAGCATC
GGTGCAGAACCAGCCGACGAACTGGCACGTGCGGCCGGTATCGCATGTGAGCGCGGCGTGGTAGTTGACGCTACGGGTGCAAG
CTCATGTCCTGCAGTATTCGCGGCAGGTGACGTAGCAGCCTGGCCCCTGAGGTCCGGTGAACTGCGCTCGCTGGAGACCTACC
TGAACAGCCACATGCAGGCTGAAACTGCCGCCGCGGCCATGTTAGGCAAGTCTATCCCGGCTCTTCAGGTGCCAACCTCTTGG
ACGGAGATTGCAGGGCATCGGATACAGATGGTTGGCGACATCGAAGGCCCCGGAGAAGTTGTCTTGCGCGGTAACGTCGAGAA
TGGTCAGCCGCTGGTGCAGTTCAGGGTTCTTGATGGTCGCGTTGAAGCCGCAACGGCTATCAATGCCCCGGAAGATTTTCCCG
TTGCAACCCGATTGGTGGCTGACCACATTCCTGTATCGGCCACAAAATTGCAGGACGCTAGCTCTAACTTGCGGGATTTTATG
AAAGCTAAAGCTGAGCGATGCGAGTGA

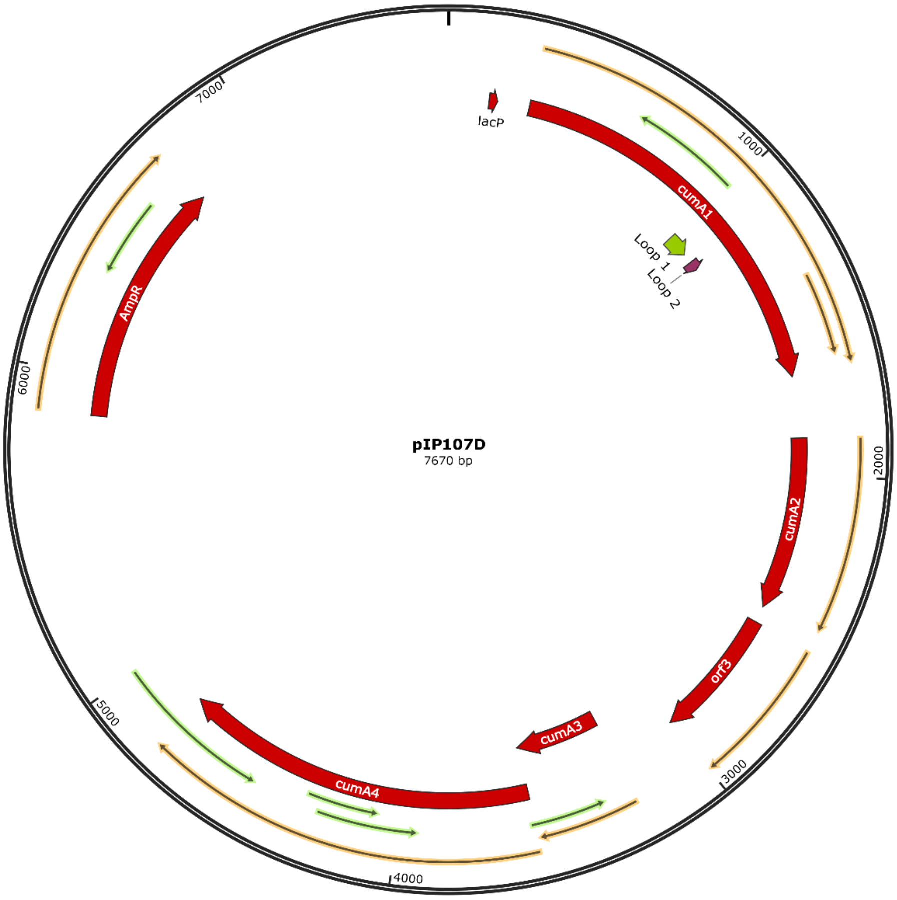

Fig. 33: Plasmid map of pIP107D.[1]

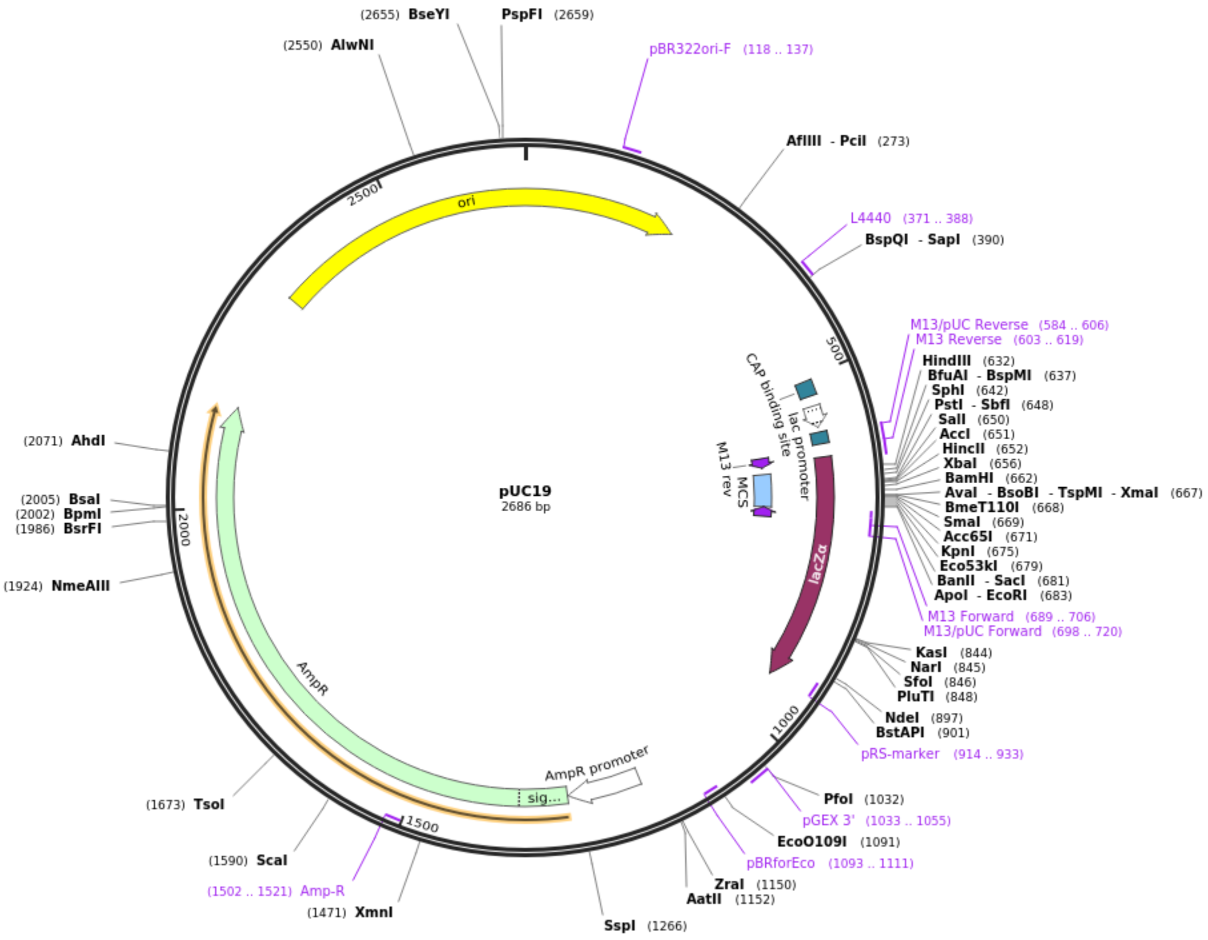

Fig. 34: Plasmid map of pUC19. (Source: https://www.addgene.org/50005, July 2020)

Supplementary References

1. Gally, C., Nestl, B. M. & Hauer, B. Engineering Rieske Non-Heme Iron Oxygenases for the Asymmetric Dihydroxylation of Alkenes. *Angew. Chemie - Int. Ed.* **54**, 12952–12956 (2015).

2. Halder, J. M., Nestl, B. M. & Hauer, B. Semirational Engineering of the Naphthalene Dioxygenase from Pseudomonas sp. NCIB 9816-4 towards Selective Asymmetric Dihydroxylation. *ChemCatChem* **10**, 178–182 (2018).

3. Vila, M. A. *et al.* Site-Directed Mutagenesis Studies on the Toluene Dioxygenase Enzymatic System: Role of Phenylalanine 366, Threonine 365 and Isoleucine 324 in the Chemo-, Regio-, and Stereoselectivity. *Adv. Synth. Catal.* **359**, 2149–2157 (2017).

4. Groeneveld, M., van Beek, H. L., Duetz, W. A. & Fraaije, M. W. Identification of a novel oxygenase capable of regiospecific hydroxylation of D-limonene into (+)-trans-carveol. *Tetrahedron* **72**, 7263–7267 (2016).

5. Boyd, D. R. *et al.* Regioselectivity and stereoselectivity of dioxygenase catalysed cis-dihydroxylation of mono- and tri-cyclic azaarene substrates. *Org. Biomol. Chem.* **6**, 3957–3966 (2008).

6. Alan, B. & Bucher, W. Menthatrienes and the Oxidation of limonene. *Helv. Chim. Acta* **53**, 770–775 (1970).

7. Scanlon, J. T. & Willis, D. E. Calculation of flame ionization detector relative response factors using the effective carbon number concept. *J. Chromatogr. Sci.* **23**, 333–340 (1985).

8. Szulejko, J. E. & Kim, K. H. Re-evaluation of effective carbon number (ECN) approach to predict response factors of 'compounds lacking authentic standards or surrogates' (CLASS) by thermal desorption analysis with GC-MS. *Analytica Chimica Acta* vol. 851 14–22 (2014).

9. Bermejo, F. A., Rico-Ferreira, R., Bamidele-Sanni, S. & García-Granda, S. Total synthesis of (+)-ampullicin and (+)-isoampullicin: Two fungal metabolites with growth regulatory activity isolated from Ampulliferina sp. 27. *J. Org. Chem.* **66**, 8287–8292 (2001).

10. Dhulut, S. *et al.* Cyclic allyl carbamates in stereoselective syn SE′ processes: Synthetic approach to sarcodictyins and eleutherobin. *European J. Org. Chem.* 5235–5243 (2007) doi:10.1002/ejoc.200700490.

11. Dong, X. *et al.* Crystal Structure of the Terminal Oxygenase Component of Cumene Dioxygenase from Pseudomonas fluorescens IP01. *J. Bacteriol.* **187**, 2483–2490 (2005).
