# Supplementary material for: Active-site loop variations adjust activity and selectivity of the cumene dioxygenase
Source: Nat Commun. 2021 Feb 17;12:1095. doi: 10.1038/s41467-021-21328-8 (PMC7889853; doi:10.1038/s41467-021-21328-8)
Supplement: Supplementary file 3 — Reporting Summary [file 41467_2021_21328_MOESM3_ESM.pdf]

nature research

Corresponding author(s): Bernhard Hauer

Last updated by author(s): Dec 21, 2020

Reporting Summary

Nature Research wishes to improve the reproducibility of the work that we publish. This form provides structure for consistency and transparency in reporting. For further information on Nature Research policies, see our Editorial Policies and the Editorial Policy Checklist.

Statistics

For all statistical analyses, confirm that the following items are present in the figure legend, table legend, main text, or Methods section.

n/a	Confirmed	
☐	☒	The exact sample size (*n*) for each experimental group/condition, given as a discrete number and unit of measurement
☐	☒	A statement on whether measurements were taken from distinct samples or whether the same sample was measured repeatedly
☒	☐	The statistical test(s) used AND whether they are one- or two-sided<br>*Only common tests should be described solely by name; describe more complex techniques in the Methods section.*
☒	☐	A description of all covariates tested
☒	☐	A description of any assumptions or corrections, such as tests of normality and adjustment for multiple comparisons
☐	☒	A full description of the statistical parameters including central tendency (e.g. means) or other basic estimates (e.g. regression coefficient) AND variation (e.g. standard deviation) or associated estimates of uncertainty (e.g. confidence intervals)
☒	☐	For null hypothesis testing, the test statistic (e.g. *F*, *t*, *r*) with confidence intervals, effect sizes, degrees of freedom and *P* value noted<br>*Give P values as exact values whenever suitable.*
☒	☐	For Bayesian analysis, information on the choice of priors and Markov chain Monte Carlo settings
☒	☐	For hierarchical and complex designs, identification of the appropriate level for tests and full reporting of outcomes
☒	☐	Estimates of effect sizes (e.g. Cohen's *d*, Pearson's *r*), indicating how they were calculated

Our web collection on statistics for biologists contains articles on many of the points above.

Software and code

Policy information about availability of computer code

Data collection	Data collection of the biotransformations was performed with standard instrument software (Agilent MSD ChemStation (Version E.02.02.1431), Agilent LC ChemStation (B.04.03.-SP1[87]), Shimadzu GCsolution (Version 2.41.00)). MD simulations were performed with YASARA (Version 16.12.29).
Data analysis	Data analysis was performed with standard instrument analysis software (Agilent MSD ChemStation (Version E.02.02.1431), Agilent LC ChemStation (B.04.03.-SP1[87]), Shimadzu GCsolution (Version 2.41.00)), YASARA (Version 16.12.29), CAVER 3.0 plugin for PyMOL (Version 2.4.0) and Excel (Version 2016). Sequence alignment with Clustal Omega online tool (Version July 2019). Primer design with SnapGene (Version 3.1.4). Phylogenetic tree visualisation with Iroki (Version October 2020). Homology modelling with SWISS-MODEL (Version January 2020). NMR analysis with Topspin (Version 3.5).

For manuscripts utilizing custom algorithms or software that are central to the research but not yet described in published literature, software must be made available to editors and reviewers. We strongly encourage code deposition in a community repository (e.g. GitHub). See the Nature Research guidelines for submitting code & software for further information.

Data

Policy information about availability of data

All manuscripts must include a data availability statement. This statement should provide the following information, where applicable:

- Accession codes, unique identifiers, or web links for publicly available datasets
- A list of figures that have associated raw data
- A description of any restrictions on data availability

All data are available from the authors upon reasonable request.

Field-specific reporting

Please select the one below that is the best fit for your research. If you are not sure, read the appropriate sections before making your selection.

- [x] Life sciences
- [ ] Behavioural & social sciences
- [ ] Ecological, evolutionary & environmental sciences

For a reference copy of the document with all sections, see nature.com/documents/nr-reporting-summary-flat.pdf

Life sciences study design

All studies must disclose on these points even when the disclosure is negative.

Sample size	No sample-size calculation was performed as the study related to the analysis of 167 protein variants either being native or containing specific mutations. Each variant was tested at least as technical triplicate to exclude variations derived from technical reasons. The sample size of technical triplicates is sufficient to evaluate strong influences of mutations on enzyme properties like product formation and distribution.
Data exclusions	No data were excluded from the analysis.
Replication	Reactions were performed in technical triplicates with standard deviations indicated and taken to verify the reproducability. Various variants were tested in biological triplicates to ensure reproducability. Biotransformation of the different substrates were repeated 7 times for the substrate styrene and 15 times for the substrates R-limonene and 2-phenylpyridine in technical triplicates. Variants with strong increases in product formation or altered regioselectivity were repeated in biological triplicates. All attempts of replication were successful.
Randomization	No animals or patients were studied. Bacterial cultures were grown under the same conditions, so randomization was not relevant.
Blinding	Blinding was not relevant as the study related to the characterization of 167 protein variants. Their identities and confirmation of specific introduced mutations were confirmed by DNA sequencing. Each protein was individually cloned, expressed and tested.

Reporting for specific materials, systems and methods

We require information from authors about some types of materials, experimental systems and methods used in many studies. Here, indicate whether each material, system or method listed is relevant to your study. If you are not sure if a list item applies to your research, read the appropriate section before selecting a response.

Materials & experimental systems

n/a	Involved in the study	
[x]	[ ]	Antibodies
[x]	[ ]	Eukaryotic cell lines
[x]	[ ]	Palaeontology and archaeology
[x]	[ ]	Animals and other organisms
[x]	[ ]	Human research participants
[x]	[ ]	Clinical data
[x]	[ ]	Dual use research of concern

Methods

n/a	Involved in the study	
[x]	[ ]	ChIP-seq
[x]	[ ]	Flow cytometry
[x]	[ ]	MRI-based neuroimaging
